# Solution‐Processed Nickel Oxide as Efficient Hole Transport Layers in Inverted Perovskite Solar Cells

**DOI:** 10.1002/advs.75257

**Published:** 2026-04-20

**Authors:** Zheng Wu, Zeliang Qiu, Yuxin Tao, Yuyang Qiu, Yuwei Duan, Jianxing Xia, Feng Li, Qiang Peng

**Affiliations:** ^1^ College of Materials and Chemistry & Chemical Engineering Chengdu University of Technology Chengdu P. R. China; ^2^ National Key Laboratory of Electronic Films and Integrated Devices School of Integrated Circuit Science and Engineering University of Electronic Science and Technology of China Chengdu P. R. China; ^3^ Institute of Molecular Plus School of Chemical Engineering and Technology Tianjin University Tianjin P. R. China; ^4^ School of Physics The University of Sydney Sydney New South Wales Australia; ^5^ School of Chemical Engineering and State Key Laboratory of Polymer Materials Engineering Sichuan University Chengdu P. R. China

**Keywords:** halide perovskites, hole transport layer, NiO_x_, solar cells, solution processing

## Abstract

Nickel oxide (NiO_x_), an efficient inorganic hole transport layer (HTL), has emerged as a key material driving the industrialization of inverted perovskite solar cells (PSCs). Its success stems from favorable energy‐level alignment, excellent charge transport features, high stability, and low cost. Among various preparation methods, solution‐based syntheses are particularly attractive for NiO_x_‐based PSCs, due to their simplicity, compatibility, and scalability. This review systematically summarizes four mainstream solution‐based techniques for synthesizing NiO_x_ HTLs, including pre‐synthesized nanoparticles, sol‐gel, solution combustion, and chemical bath depositions. For each method, we discuss the reaction mechanisms and processing features, the resulting film properties, and strategies for performance optimization in PSCs. We then outline challenges associated with each route and highlight recent advances in material modification, process engineering, and structural design aimed at overcoming these bottlenecks and enhancing device efficiency. Further research should address critical issues such as uniformity control in large‐area, rapid synthesis processes and reliable preparation of high‐crystallinity, low‐defect films at low temperatures. A deeper understanding of the relationships among solution processes, microstructure, and device performance will be essential for fully realizing the potential of solution‐based NiO_x_ HTLs in high‐performance, high‐stability, and low‐cost perovskite photovoltaic technologies.

## Graphical abstract

This review summarizes recent advances in four mainstream solution‐based techniques for synthesizing NiO_x_ hole‐transport layers for high‐performance perovskite photovoltaic cells, and highlights the key challenges and future prospects for achieving high efficiency, long‐term stability, and low‐cost perovskite photovoltaic technologies toward commercialization.

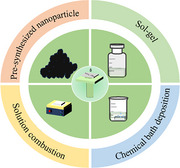

## 1 Introduction

As a representative of next‐generation photovoltaic technologies, halide perovskite solar cells (PSCs) have made remarkable progress over the past decade. Their power conversion efficiency (PCE) has increased from 3.8% to over 27%, approaching the theoretical limit of monocrystalline silicon solar cells [1, 2, 3]. To date, the certified PCE of single‐junction perovskite solar cells has reached 27.2%, while all‐perovskite tandem cells have achieved a certified stabilized efficiency of up to 30.1% [3, 4, 5, 6]. Such the remarkable improvement in their PCE values is primarily attributed to the unique optoelectronic properties of perovskite active materials, mainly including their high optical absorption coefficient (>10^4^ cm^−1^), long carrier diffusion length (>1 µm), and their tunable bandgap (1.2–2.3 eV) [1, 7], along with the promising structure design of the relevant photovoltaic devices. In particular, among various device designs of perovskite solar cells, inverted structure (*p‐i‐n*) PSCs exhibit excellent performance, enhanced stability, and strong process compatibility, making them one type of the most commercially promising technical pathways [8]. However, scaling up from small‐area lab‐based *p‐i‐n* devices (<0.1 cm^2^) to large‐area modules (>100 cm^2^) causes significant efficiency losses, with PCE dropping from >25% to <20%, which remains a major bottleneck for their industrialization [9]. Another particularly prominent challenge is the relatively high cost of the whole set of cells. Notably, the cost of the raw materials for synthesizing perovskite materials is about 1/20 that of traditional materials like crystalline silicon. Also, the perovskite active layers can be normally fabricated via low‐cost solution processing methods, while crystalline silicon for solar cells normally requires complex instruments with highly accurate control. However, the whole set of perovskite devices also includes other key functional layers, in particular the HTLs, which still rely on the expensive raw materials and the complex experimental processes [8]. For example, conventional organic HTL materials such as Spiro‐OMeTAD cost up to USD500/g, and normally require hygroscopic dopants (e.g., lithium bis(trifluoromethanesulfonyl)imide, Li‐TFSI), which would also accelerate the degradation of photovoltaic device performance [10, 11]. In contrast, inorganic hole transport materials such as metal oxides could offer promising features for promoting perovskite photovoltaic cell performance and stability. Among various metal oxides, NiO_x_ could offer excellent charge transport properties, promising chemical stability, high visible‐light transmittance (>80%), and well‐matched energy levels with widely ranged perovskite materials (valence band maximum (VBM) of NiO_x_ is about 5.3 eV) [7, 12, 13, 14]. Furthermore, the cost of the raw materials for the synthesis of NiO_x_ (USD10–20/g) is significantly lower than that of organic HTMs and most of other metal oxides, making NiO_x_ the ideal HTL candidate for high‐performance PSCs [15, 16]. Taken together, *p‐i‐n* PSCs have been widely identified as one of the most commercially promising technical routes; meanwhile, solution‐processed NiO_x_ has emerged as an ideal HTL candidate with the characteristics of low cost, facile synthesis, high stability, and well‐matched energy levels. The combination of these two advantages would become a crucial research direction for breaking through the bottlenecks in the industrialization of PSCs. To gain a deeper understanding of the value of such a combination, it is essential to first clarify the specific position of NiO_x_ HTLs in *p‐i‐n* PSC devices and the core functions they undertake, which is exactly a key link in ensuring PSC device performance and stability for real‐world applications.

In the *p‐i‐n* PSC devices, NiO_x_ HTLs are positioned at the top of the devices, directly exposed to ambient conditions. As such, NiO_x_ layers could serve multiple critical functions and roles. First, NiO_x_ layer can work as an efficient HTL, requiring the capability of efficiently extracting and transporting photo‐generated holes from the perovskite active layer. Promisingly, NiO_x_ has been demonstrated to exhibit high electrical conductivity (>10^−3^ S cm^−1^) and a matched valence band (VB) position (∼5.3 eV) with that of the perovskite layer, ensuring that it can serve as the effective HTL. Second, NiO_x_ layer, as an ideal HTL, should effectively block the electron recombination at the electrode interface, thus reducing the interfacial energy losses. Plus, NiO_x_ layer can also function as an environmental barrier layer to the ambient conditions, which means that it should protect the underlying perovskite active layer from moisture and oxygen corrosion [17]. Notably, a great amount of research has indicated that NiO_x_ HTLs can be prepared via diverse methods. To reduce the overall device costs, more researchers have developed diverse synthesis processes for synthesizing NiO_x_ layers, in particular the solution‐based approaches. These solution‐processed approaches could significantly lower the cost of the NiO_x_ HTLs through aspects such as simplifying fabrication procedures, lowering reaction temperatures, adopting low‐cost precursors, and minimizing equipment dependency [18, 19, 20]. Most recent studies have shown that the PSCs based on the optimized, solution‐processed NiO_x_ could exhibit remarkable device performance and excellent long‐term stability (>1200 h at 85°C at AM1.5G illumination), significantly surpassing the devices based on organic HTLs (<500 h) [11, 21, 22]. More significantly, by using solution‐processed NiO_x_ as the HTL, the certified PCE of single‐junction solar cells has reached up to 27.2%, surpassing that of devices with the same architecture employing a pure self‐assembled monolayer (SAM) HTL (achieving a certified efficiency of 27.17%); meanwhile, all‐perovskite tandem solar cells have demonstrated an impressive certified efficiency as high as 30.1% [3, 4, 5]. Owing to the significant advantages, including high charge transport properties, suitable band structures, high stability, low costs, simple equipment requirements, and scalability, solution‐processed NiO_x_ films working as HTLs have emerged as a promising technological pathway toward high‐efficiency and high‐stability PSCs, further advancing their commercialization.

This review systematically summarizes four mainstream solution‐processing techniques for the synthesis of NiO_x_ HTLs, including pre‐synthesized NiO_x_ nanoparticle (NP) inks, sol‐gel methods, solution combustion synthesis, and chemical bath deposition (CBD). We focus on discussing the process characteristics and underlying working mechanisms of these methods, pointing out the existing challenges for each method, and integrating improvement strategies for key properties of the obtained NiO_x_ HTLs. We further clarify how the variations in the properties of NiO_x_ HTLs could affect the performance and stability of the relevant PSCs. Significantly, these four types of methods enable the deposition of high‐quality NiO_x_ HTLs through facile liquid‐phase chemical reactions, effectively overcoming the limitations of the traditional deposition processes, such as vacuum sputtering and high‐temperature annealing, which normally require high energy consumption and inherent equipment complexity. By systematically reviewing and providing perspectives on these low‐cost, scalable solution‐processing techniques, this review aims to offer both the fundamental insights and practical guidance for advancing the commercialization of high‐performance, highly stable NiO_x_‐based PSCs.

## 2 Perovskite‐Based Photovoltaics

### 2.1 Promising Features of Halide Perovskites

#### 2.1.1 Structural Properties

**FIGURE 1:**
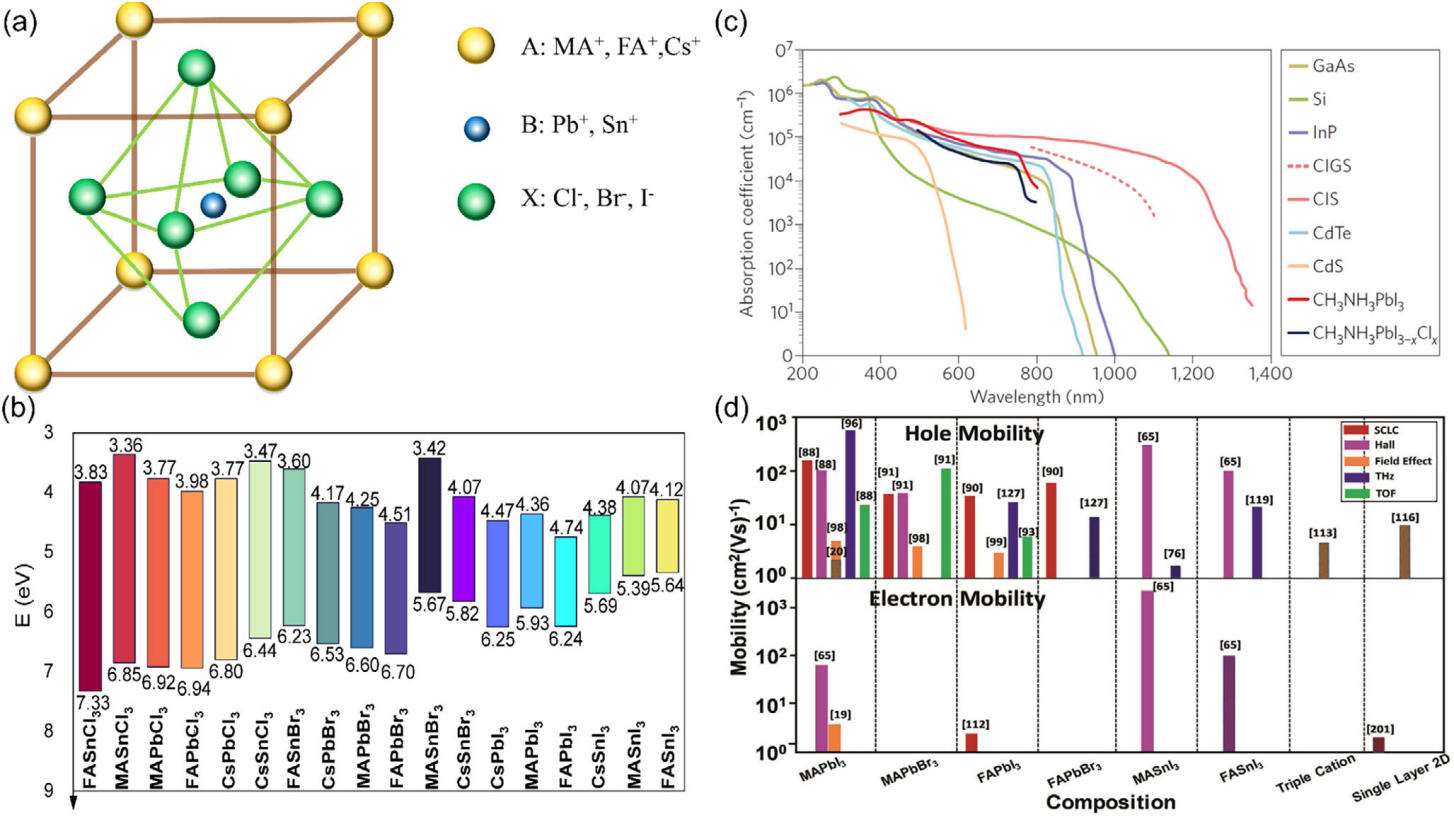
(a) Structural schematic diagram of perovskite materials. (b) Band structure of different types of perovskite materials. (c) Absorption coefficient of typical perovskites MAPbI_3_ and MAPbI_3‐x_Cl_x_ compared with other photovoltaic active materials. Reproduced with permission [31]. Copyright 2014, Springer Nature Limited. (d) Charge‐carrier mobility values of a few representative hybrid perovskites determined using different techniques (upper panel: hole mobility; lower panel: electron mobility). Reproduced with permission [32]. Copyright 2021, Wiley‐VCH.

Halide perovskites, as the core light‐absorbing materials for the third‐generation photovoltaic technology, possess excellent optoelectronic properties endowed by their unique crystal structures. The unique structural and promising optoelectronic properties make this class of materials a key driver for breaking through photovoltaic efficiency limits. Halide perovskite materials adhere to the typical ABX_3_‐type perovskite crystal structure. The A site is generally occupied by monovalent cations, including organic cations such as methylammonium cation (MA^+^) and formamidine cation (FA^+^), or inorganic metallic cations like Cs^+^, of which the formed materials can be normally referred to as the organic–inorganic hybrid and all‐inorganic perovskites. The B site is a divalent metal cation; traditionally, it can be Pb^2+^, Sn^2+^ (serving as the focus in Pb^2+^ replacement research), or their mixture. The X site consists of halogen anions, including I^−^, Br^−^, and Cl^−,^ or their mixture (Figure 1a) [23]. In this structure, the A‐site cation occupies the body‐centered position of the perovskite lattice. The B‐site cation forms a BX_6_ octahedron with six surrounding X‐site anions, and these octahedrons form a three‐dimensional network structure through corner‐sharing connections, providing continuous channels for charge carrier transport [23]. Notably, the ionic radius ratio between A‐site and B‐site cations exerts a significant influence on the lattice stability of the formed halide perovskites, which can be evaluated using the Goldschmidt tolerance factor (*t*) and octahedral factor (*µ*) [24]. When the *t* value falls within the range of 0.8–1.0 and the *µ* value is in the range of 0.414–0.732, the related halide perovskites tend to form a stable cubic or tetragonal phase featuring excellent physical properties; if these two values are beyond the above‐mentioned ranges, it may lead to the lattice distortion or failure to form the typical and stable perovskite structures [25].

#### 2.1.2 Composition Diversity and Bandgap Tunability

On the basis of the above‐mentioned *t* and *µ* factors for evaluating the structure stability, there has emerged a series of halide perovskites that have been developed for realizing tunable properties and versatile functionalities (Figure 1b). The exceptional tunability is prominently reflected in their optical absorption, which can be widely adjusted by regulating the chemical composition. For instance, changing the proportion of halogens at the X site, such as replacing part of I^−^ with Br^−^, is a widely used approach for regulating the perovskite bandgap [26]. Furthermore, adjusting the A‐site cations, such as partially substituting FA^+^ with smaller cations like Cs^+^ or MA^+^, can not only optimize the band gap but also enhance lattice stability by reducing the tolerance factor and formation enthalpy [27]. This is exemplified by the distinct bandgaps of foundational perovskites, with MAPbI_3_, FAPbI_3_, and CsPbI_3_ being about 1.59, 1.51, and 1.72 eV, respectively [28]. This compositional flexibility, enabling precise bandgap engineering from the visible to the near infrared, is foundational for advanced energy and other optoelectronic device applications.

#### 2.1.3 Optical Properties

From the perspective of optical properties, halide perovskites exhibit an extremely high light absorption coefficient, which is one of their core advantages to serve as the high‐efficiency light‐absorbing layers for photovoltaic devices. Notably, a typical perovskite film like MAPbI_3_ with a thickness of tens to hundreds of nanometers could achieve effective light absorption, reaching up to 10^5^ cm^−1^, across the visible to near‐infrared region in the solar spectrum (Figure 1c), greatly reducing material consumption and device fabrication costs [29]. Furthermore, the band gap tunability of perovskites, as exemplified by materials like FAPbI_3_ with a bandgap of ∼1.51 eV [28], makes them suitable not only for single‐junction photovoltaic devices but also for ideal pairing with other photovoltaic technologies such as silicon‐based and copper indium gallium selenide (CIGS) technologies, thereby constructing high‐efficiency tandem photovoltaic devices [30].

#### 2.1.4 Charge Transport Properties

Charge carrier dynamic properties represent another key advantage of halide perovskites, which distinguish them from traditional photovoltaic materials and make them promising for realizing high‐performance photovoltaic cells. Halide perovskites exhibit relatively low exciton binding energy, which is even smaller than or comparable to the thermal energy at room temperature. This implies that photoexcited excitons can dissociate spontaneously into free electrons and holes, enabling efficient carrier separation without the need for additional energy input [33]. On the other hand, various halide perovskites with high quality could exhibit high charge carrier mobility values (Figure 1d), with the carrier diffusion lengths exceeding 1 µm [32, 34, 35]. Some optimized perovskite materials can even achieve a diffusion length of 2–3 µm [29, 32, 34, 35]. Coupled with the long carrier lifetimes, the long diffusion lengths of perovskite materials ensure that the photoexcited carriers can travel to the electrodes with minimal losses, thereby underpinning high device efficiencies [36].

Beyond the widely studied three‐dimensional (3D) perovskites, their low‐dimensional (LD) counterparts (e.g., two‐dimensional (2D) and one‐dimensional (1D) phases) have emerged as a crucial material family for interfacial engineering. By incorporating bulky organic ammonium cations, the inorganic octahedral frameworks in LD perovskites are spatially confined, which often results in enhanced ambient stability and distinct optoelectronic properties. In photovoltaic devices, LD perovskites are frequently employed as passivating capping layers atop 3D perovskite films to form 3D/LD heterostructures. This approach effectively suppresses non‐radiative recombination and ion migration at the surface, thereby concurrently improving device efficiency and operational stability [37]. The design toolbox for such LD layers has been greatly expanded, for example, through novel synthesis methods that allow the incorporation of diverse metal cations beyond Pb^2+^, enabling fine‐tuning of interface energetics and stability [38].

In addition, halide perovskites also possess excellent defect tolerance—even if there is a certain density of defects in the perovskite films (such as vacancies and anti‐site defects), their carrier recombination rate can remain at a low level. This characteristic reduces the extreme requirements for the crystalline quality of the films and provides convenience for large‐scale fabrication of PSCs [39].

#### 2.1.5 Other Advantages

Compared with traditional photovoltaic active materials (such as Si, copper indium gallium selenide (CIGS)), perovskite materials show significant advantages in raw material cost and synthesis processing, which are also the key reasons why this class of materials becomes the core candidates for the next generation of photovoltaic technology. From the perspective of raw materials, the chemical composition of perovskite involves low‐cost and readily available chemicals. For example, A‐site MA^+^ and FA^+^ can be prepared by simple organic synthesis, and their costs are much lower than that of high‐purity silicon materials; the metal ions such as Pb^2+^ and Sn^2+^ at the B‐site are derived from common halide salts (such as PbI_2_ and SnI_2_), and do not require rare metals or high purity extraction; the X‐site anions come from halide salts with low costs, and only tens to hundreds of nanometers thick films are needed to meet the light absorption requirements, greatly reducing the consumption of raw materials [40]. In contrast, Si‐based solar cells rely on high‐purity polysilicon with a purity of more than 99.9999%, and the purification process has high energy consumption and high cost. CIGS is dependent on rare metals such as indium and gallium, which are significantly affected by resource reserves and price fluctuations, and it is difficult to achieve low‐cost scaling. More importantly, perovskite layers have excellent solution processability. Perovskite precursors can be prepared by a simple dissolution process. Organic cationic iodide salts (such as MAI, FAI) and metal halides can be simply dissolved in the conventional polar solvents such as N,N’‐dimethylformamide (DMF) and dimethyl sulfoxide (DMSO) to form a uniform and stable precursor solution without complex processes such as high temperature melting or vacuum sputtering [29]. The subsequent film‐forming process can use low‐cost solution processes such as spin coating, slit coating, spray pyrolysis, etc., and the film‐forming temperature is far lower than the annealing temperature of silicon‐based materials and the deposition temperature of CIGS, without high‐energy high‐temperature equipment [1].

### 2.2 Design of Perovskite Solar Cells

**FIGURE 2:**
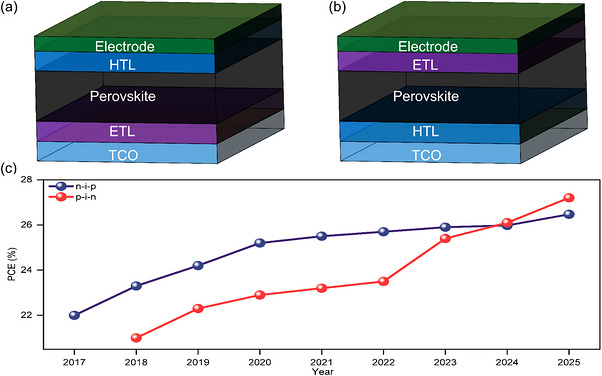
Typical structures of perovskite solar cells: (a) normal *n‐i‐p* structure and (b) inverted *p‐i‐n* structure. (c) Notable efficiency evolution of single‐junction *n‐i‐p* and *p‐i‐n* perovskite solar cells since 2017. Reproduced with permission [5, 43, 44, 45, 46]. Copyright 2024, Springer Nature Limited.

The core design of halide perovskite solar cells lies in establishing a complete pathway of light absorption, photo‐induced carrier separation, charge carrier transport, and charge carrier collection. The device structure should satisfy the synergistic effect of each involved functional layer to maximize light utilization efficiency and carrier extraction and transport efficiency. From the perspective of overall architecture, perovskite solar cells generally include a substrate/electrode, hole (electron) transport layer, perovskite active layer, electron (hole) transport layer, and another electrode. In general, they can be mainly divided into two categories: the normal structure (*n‐i‐p*) and the inverted structure (*p‐i‐n*) (Figure 2a,b). The core difference between these two device structures lies in the deposition order of the electron transport layer (ETL) and the HTL, and device characteristics derived therefrom [41].

The normal *n‐i‐p* device structure is the earliest developed architecture for perovskite solar cells, with a typical layer sequence that includes glass integrated with transparent conductive oxide (TCO, e.g., FTO or ITO), ETL, perovskite absorption layer, HTL, and metal electrode (e.g., Au, Ag, or Al; Figure 2a). In this structure, the ETL is usually a metal oxide (e.g., TiO_2_ or SnO_2_). Its main functionality is to extract photo‐excited electrons from the conduction band (CB) of the perovskite absorption layer, to transport these electrons to the TCO electrode, and at the same time, to block the transport of holes toward the electrode to suppress the recombination of carriers. The perovskite absorption layer is located between the ETL and HTL, serving as the core part for light absorption and generation of charge carriers. Its thickness is usually controlled to be within the range of 300–600 nm, aiming to balance light absorption efficiency and carrier transport distance [42]. The HTL layer is responsible for extracting holes from the VB of the perovskite and transporting them to the metal electrode.

In contrast, the inverted *p‐i‐n* device structure has a layer sequence that comprises TCO, HTL, perovskite absorption layer, ETL, and metal electrode (Figure 2b). It was originally designed to address the issues of high‐temperature fabrication of oxide ETL (for example, TiO_2_ requires annealing at a high temperature of 450°C) and poor stability of organic HTLs in the *n‐i‐p* normal structure. In this structure, the HTLs are the typical materials, such as NiO_x_, PEDOT:PSS, and PTAA, which can be prepared at relatively low temperatures and can form high‐quality films without further high‐temperature treatment. These advantages could make them compatible with flexible substrates, thus being beneficial for the fabrication of flexible and large‐sized photovoltaic solar cells. Furthermore, in this structure, the ETL are mostly the organic semiconductors (e.g., PCBM, C_60_) or metal oxides (e.g., SnO_2_), which can also be prepared at relatively low temperatures. Notably, these materials not only have appropriate conduction band (CB) energy levels but can also further suppress carrier recombination through interface modification. Compared with the normal *n‐i‐p* structure, there are also some other advantages for the inverted *p‐i‐n* structured devices; a notable advantage of them lies in their low current–voltage (*J–V*) hysteresis effect [47].

### 2.3 Emerging Inverted Perovskite Solar Cells

Perovskite solar cells with the inverted *p‐i‐n* structure, featuring a low‐temperature fabrication process, low *J–V* hysteresis effect, and excellent compatibility, have become a crucial direction for the scaling‐up and industrialization of perovskite photovoltaic technology (Figure 2c). Compared with the normal *n‐i‐p* structure, the core advantages of the inverted device structure stem from the synergistic optimization of its layer sequence design and material selection; this enables this device structure to exhibit unique potential in flexible devices and tandem devices with high performance and long‐term stability [48].

From the perspective of fabrication processes, the most prominent feature of the inverted *p‐i‐n* structure is relatively low‐temperature requirements throughout the fabrication process, in which all the involved functional layers, including HTL, perovskite layer, and ETL, can be prepared at temperatures below 300°C, with some materials even being deposited at room temperature. In detail, the perovskite absorption layer can achieve high‐quality crystallization via solution‐based methods, such as spin coating, blade coating, and slot‐die coating, at temperatures ranging from room temperature to 150°C. As for the ETLs (normally, the organic and metal oxide semiconductors), they can be deposited at low temperatures through solution‐based spin coating or vacuum evaporation. This low‐temperature process not only reduces the equipment energy consumption and production costs but also avoids the damage of flexible substrates caused by high temperatures, thus making the inverted structure the only viable architecture for flexible perovskite solar cells [49].

As stated above, low *J–V* hysteresis effect is another core advantage of the inverted PSC devices, and this characteristic stems from its reverse design for the charge carrier transport and the ion migration directions. In the normal *n‐i‐p* structure, photo‐excited electrons are transported toward the TCO, while ions in the perovskite layer tend to migrate toward and accumulate at the HTL or ETL under bias voltage; this leads to significant differences in the *J–V* curves between the forward and reverse scans. In the inverted devices, however, photo‐excited holes can transport toward the TCO while electrons toward the metal electrode; the ion migration direction is opposite to the carrier transport direction, which can effectively suppress ion accumulation [50]. Furthermore, the interfaces related to the inorganic HTL and organic ETL in this device structure exhibit high stability and are less prone to ion exchange with the perovskite active layer, further reducing the hysteresis effect. Studies have shown that the hysteresis rate of the optimized inverted perovskite solar cells can be less than 2%, and some devices even achieved the hysteresis‐free performance; this is crucial for the measurement of actual power output and the real‐world application of photovoltaic devices [51].

In terms of stability, the inverted structure could be beneficial for significantly enhancing the long‐term operational lifetime of the devices through material selection and interface design. In the normal *n‐i‐p* structure, the dopants (such as lithium bis(trifluoromethanesulfonyl)imide (LiTFSI) and 4‐*tert*‐butylpyridine (TBP)) of the organic HTLs exhibit hygroscopicity, which tends to cause device degradation. By contrast, the inorganic and organic HTLs commonly used in the inverted structure require no doping or only a small amount of doping, which can reduce moisture‐induced degradation on the fabricated photovoltaic devices [52]. Meanwhile, the ETLs in the devices with the inverted structure can act as the protective layer to avoid direct contact between oxygen and the perovskite layers. Studies have shown that the encapsulated inverted perovskite solar cells could operate stably for more than 1000 h under a nitrogen atmosphere (85°C) while retaining over 80% of their initial efficiency [53]. Most recent research showed that, under ambient conditions, the optimized devices with inverted structure treated with interface passivation (e.g., SAMs) could maintain stable operation for over 500 h [48].

From the perspective of device scaling‐up, the inverted structure is more suitable for large‐area fabrication processes as compared to the normal device structure. In the *n‐i‐p* structured devices, the high‐temperature fabrications of ETLs and the relatively low uniformity of organic HTLs through the solution processes would limit the realization of large‐scale perovskite solar cells. By contrast, all the involved layers in the inverted devices can be prepared via scalable solution‐based methods, such as slot‐die coating and blade coating. In addition, the low‐temperature process has lower requirements for the temperature uniformity of large‐area substrates, which would also be helpful for achieving the large‐scale perovskite photovoltaic devices with both high performance and high yield [54]. This scaling‐up potential has positioned the inverted device structure as the core candidate architecture for the future industrialization of perovskite photovoltaic technologies.

## 3 Advanced Features of NiO_x_ for Hole Transport

### 3.1 Structural Properties of NiO_x_

On the basis of the above discussions, the inverted‐structured perovskite photovoltaic devices have unique advantages over the normal‐structured devices, holding the great promise for the realization of cost‐efficient, large‐scale, high‐performance/stability, and environmentally friendly halide perovskite solar cells, accelerating their commercialization. For this, employing suitable materials as HTLs would be a critical step. Recently published works showed that solution‐processed NiO_x_ has attracted more attention to work as a suitable HTL layer for highly efficient inverted perovskite solar cells. This can be attributed to the advantageous properties of solution‐based NiO_x_ layers, including their remarkable structural and physical properties.

**FIGURE 3:**
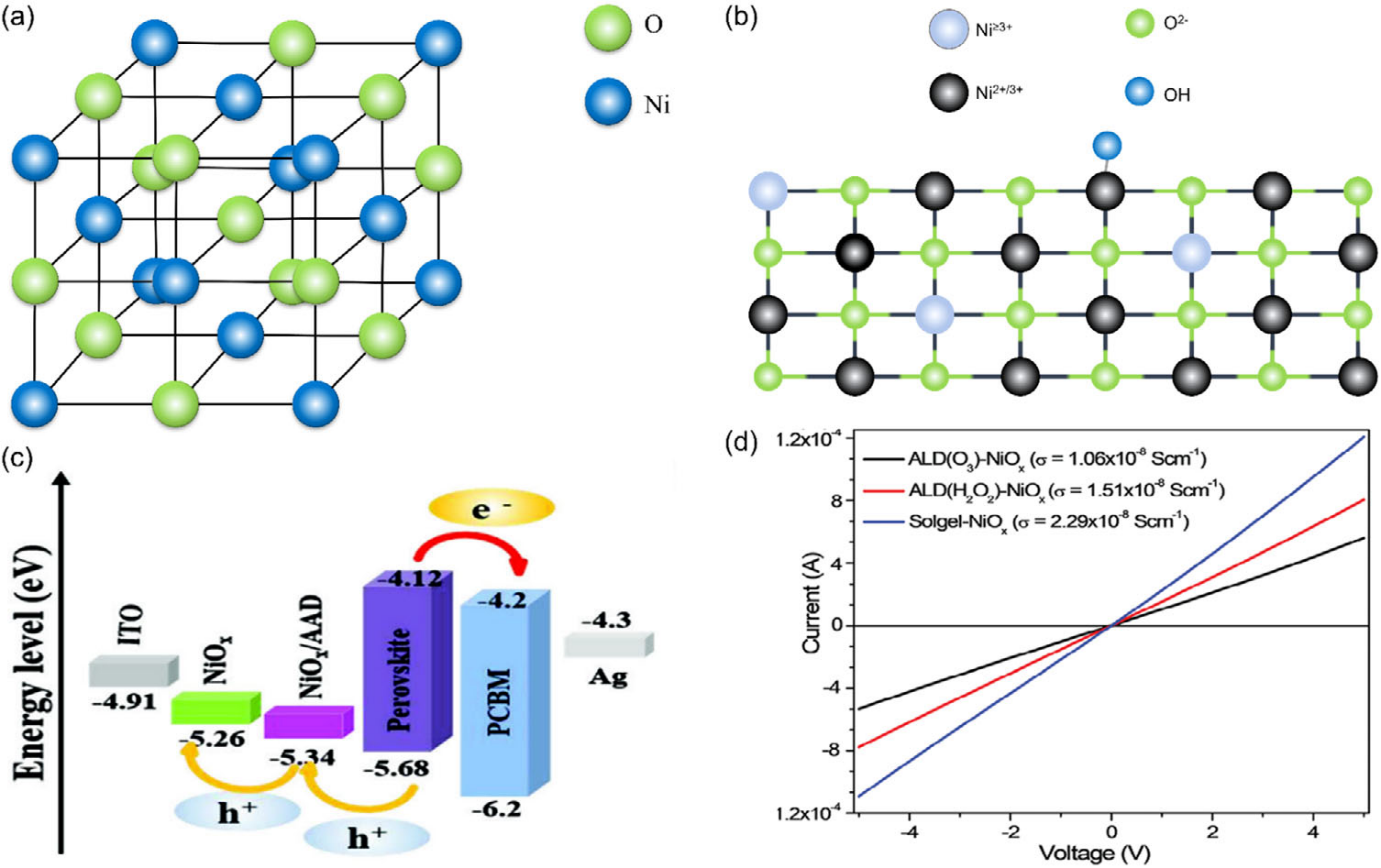
(a) The crystal structure of NiO_x_. (b) The valence state of Ni cations within NiO_x_ films. (c) The VBM values of NiO_x_ thin films w/wo 3‐amino‐1‐adamantanol (AAD) (‐5.26 and ‐5.34 eV, respectively). Reproduced with permission [14]. Copyright 2023, Wiley‐VCH. (d) Current–voltage (*I–V*) curves of NiO_x_ films deposited via O_3_‐driven ALD, H_2_O_2_‐driven ALD, and sol–gel method. Reproduced with permission [63]. Copyright 2024, Wiley‐VCH.

In regard to the structural properties of solution‐processed NiO_x_ films, mainly including their crystal structure, as well as the morphology and defect structure of the formed films, which would directly determine their hole transport efficiency and interfacial compatibility with other layers in the devices. From the perspective of their crystal structure, NiO_x_ typically exhibits a rock‐salt (NaCl‐type) crystal structure with a space group of Fm‐3m (Figure 3a) [21]. In this structure, Ni ions occupy the vertices and face‐centered positions of the face‐centered cubic lattice, while O ions occupy the body‐centered and edge‐centered positions [55]. In NiO with an ideal stoichiometric ratio, Ni exists in the +2‐oxidation state (Ni^2+^) and O in the −2‐oxidation state (O^2−^), of which the lattice constant is approximately 4.17 Å. However, as for the synthesized NiO_x_ thin films, the stoichiometric ratio usually deviates from 1, due to the presence of excess O or Ni vacancies. Additionally, Ni^3+^ cations (and even a small amount of Ni^4+^ cations) may form, leading to the formation of non‐stoichiometric NiO_x_ films (Figure 3b) [18, 56, 57]. Such a non‐stoichiometric property could serve as the fundamental reason for NiO_x_ thin films, functioning as the *p*‐type semiconductor for transporting holes efficiently, while the formation of a small amount of Ni^3+^ cations can further enhance the hole concentration. These factors together determine the promising electrical properties of NiO_x_ thin films, beneficial for the hole transport within the fabricated photovoltaic devices [58]. It is worth noting that the crystal structure and defect characteristics of NiO_x_ are the core guarantee for its excellent stability. NiO_x_ has a stable rock‐salt type lattice structure, and the non‐metrically balanced formation of Ni^3+^ (Ni_2_O_3_, NiOOH) enhances its chemical inertness, making it less prone to harmful redox reactions with the perovskite layer and avoiding problems such as acidic corrosion of organic HTL (such as PEDOT:PSS) and the hydrophobic film defect of PTAA [59, 60]. This structural stability not only improves the environmental tolerance of the NiO_x_ film itself (resistance to moisture, heat, and UV), but also further inhibits carrier recombination and interface degradation through defect passivation (such as ion doping, interface modification), laying the foundation for the long‐term stable operation of the device [61, 62].

The defect structure is a key structural factor affecting the electrical properties of NiO_x_ thin films and thus the performance of the related energy and optoelectronic devices; these defects mainly include Ni vacancies (*V*_Ni_), oxygen vacancies (*V*_O_), and interface defects [64]. Ni vacancies are the most dominant intrinsic defects in NiO_x_ thin films, and the concentration of *V*_Ni_ directly determines the hole concentration and electrical conductivity of the formed NiO_x_ films. An optimal range of vacancy concentrations can yield suitable electrical conductivity values that meet the requirements for efficient hole transport in devices such as solar cells. However, an excessively high density of these vacancies is detrimental, as it intensifies charge carrier scattering, which in turn leads to a significant reduction in carrier mobility and overall device performance [65]. Oxygen vacancies typically form under oxygen‐deficient conditions and can introduce donor levels, which exert adverse effects on *p*‐type conductivity of the films. Therefore, it is necessary to control the oxygen atmosphere during the fabrication process to reduce the concentration of *V*_O_ [10, 66, 67]. Interface defects mainly exist at the interfaces between NiO_x_ films and TCO layers and/or perovskite active layers, including the uncoordinated Ni ions, hydroxyl groups, and adsorbed water; these defects can cause interface carrier recombination, which can be passivated through effective interface modification (these will be detailed in the following sections) [59].

### 3.2 Electrical Features of NiO_x_

The excellent electrical properties of NiO_x_ thin films, including their promising *p*‐type semiconducting property, high carrier transport efficiency, high conductivity, and suitable energy‐level alignments, serve as the core foundation for ensuring the efficient extraction and transport of holes in the related photovoltaic devices. More significantly, these properties could be interconnected and function synergistically, jointly determining the hole transport performance of NiO_x_ layers in the fabricated photovoltaic devices; meanwhile, they can also be precisely regulated through material design and process optimization.

As stated above, the *p*‐type semiconducting transport features of NiO_x_ thin films highly depend on their defect types and levels. Regarding their electrical conductivity, which is also a key parameter for evaluating the hole transport capability of NiO_x_ thin films, the magnitude would directly affect the series resistance and fill factor (*FF*) of the fabricated photovoltaic devices. The intrinsic electrical conductivity of NiO_x_ is normally low, which fails to meet the requirements for efficient hole transport. Encouragingly, by regulating the fabrication processes and employing suitable doping engineering, the electrical conductivity of NiO_x_ thin films can be highly increased for highly efficient hole transport in the high‐performance perovskite photovoltaic devices [68].

The band gap of NiO_x_ (normally, *E*_g_ ∼ 4 eV) determines the hole extraction barrier between NiO_x_ thin films and the perovskite absorption layers, serving as a core factor that affects hole extraction efficiency [17]. What is more, NiO_x_ can effectively absorb ultraviolet light, which reduces the direct irradiation of UV on the perovskite layer and thus alleviates UV‐induced degradation of the perovskite active layer [69]. Under ideal conditions, the VBM of NiO_x_ thin films should be close to or slightly higher than that of the perovskite active layers to ensure that photo‐induced holes can be spontaneously injected from the perovskite layers into NiO_x_ films. Meanwhile, the conduction band minimum (CBM) of NiO_x_ films should be much higher than that of the perovskite layers to block electron transport toward NiO_x_ films and avoid the charge carrier recombination. The optimization of energy level alignment can significantly improve the hole extraction efficiency. Studies have shown that the hole extraction efficiency can reach up to 90% when the VBM offset between NiO_x_ films (∼5.3 eV) and the typical perovskite layers is less than 0.3 eV (Figure 3c); while an obvious hole extraction barrier would occur if the offset is greater than 0.5 eV, leading to the increased charge carrier recombination and thus the reduced photovoltaic device performance [70]. In addition, the VBM level of NiO_x_ thin films is also well‐matched with the work function (*W*_F_) of normally used metal electrodes (e.g., Au or Cu), which could be beneficial for the collection of the photo‐induced holes transferred from the perovskite active layers and passed through NiO HTLs.

Carrier transport efficiency is a comprehensive reflection of the electrical property of NiO_x_ thin films, and it depends primarily on their carrier mobility, carrier lifetime, and interface transport resistance. NiO_x_ thin films with high crystallization and suitable defect levels possess high carrier mobility (2.53 cm^2^ V^−1^ s^−1^) and high conductivity, where NiO_x_ films made by the sol‐gel method achieved a high conductivity of 2.29 × 10^−8^ S cm^−1^, as demonstrated in Figure 3d [71]. Ensuring their excellent carrier transport efficiency to satisfy the photovoltaic device requirements by combining their excellent film compactness and interface contact quality [17, 72]. Carrier lifetime is a parameter that measures the survival time of charge carriers in NiO_x_ thin films. In general, the carrier lifetime of the pure NiO_x_ thin films is approximately 10–100 ns, which can be further extended to 100 – 500 ns through suitable doping engineering and interface passivation treatments [73].

### 3.3 Other Characters: Low‐Cost and Facile Synthesis Process

In general, NiO_x_ thin films can be synthesized via various methods, including thermal deposition, sputtering, and low‐cost solution‐based approaches. Notably, solution‐processed techniques have attracted significant attention due to their low cost, low energy consumption, and their ability to produce high‐quality thin films comparable to those made by other methods.

In contrast, the traditional processes normally require high‐cost raw materials, complex equipment, and precise operations. For example, the pulsed laser deposition (PLD) process, a key physical vapor deposition technique for high‐quality NiO_x_ films, relies on the high‐purity NiO targets sintered from high‐grade NiO powder to prevent film defects. For Li‐doped NiO_x_ thin films (that is, NiO:Li_x_ used as hole transport layers in perovskite solar cells), the customized targets with precise Li ratios are required, which increases raw material cost and operation complexity. Additionally, the targets with particular sizes used in the PLD process normally have limited utilization, only a small part of the target is ablated, further increasing the expense. Similarly, plasma‐assisted molecular beam epitaxy (PAMBE) for synthesizing NiO_x_ thin films requires high‐purity Ni metal to maintain a stable Ni flux and avoid impurities that would degrade the thin film quality. During the growth process, NiO_x_ thin films can be deposited at the orifice of the Ni effusion cell, reducing flux stability and necessitating frequent maintenance or replacement, which would also increase the cost. In addition, thermal evaporation and sputtering processes also follow this pattern, requiring high‐purity Ni or NiO_x_ sources to ensure the quality of the obtained NiO_x_ thin films, thereby causing high costs. Beyond consumables such as targets and evaporation materials, these vacuum‐based methods also involve substantial initial investments in equipment and supporting resources such as high‐purity protective gases.

In comparison, the solution‐based methods employ low‐cost nickel salts, such as nickel nitrate (Ni(NO_3_)_2_), as the core precursors, along with small amounts of solvents, bases, or fuels (e.g., deionized water, ethylene glycol, or ethylenediamine). In addition, the solution‐based methods do not require complex equipment and additional sources like particular gases. These make solution‐processed NiO_x_ thin films with high‐quality, cost‐effective, and high‐efficiency alternatives.

Below, we describe in detail the four types of solution‐based methods developed so far for the synthesis of high‐quality NiO_x_ thin films that could serve as efficient HTLs in high‐performance and low‐cost halide perovskite photovoltaic cells.

## 4 Solution‐Processed NiO_x_ as HTLs in PSCs

### 4.1 Pre‐Synthesized Nanoparticle (NP) Method

#### 4.1.1 Experimental Process and Mechanism

The pre‐synthesized NiO_x_ nanoparticle (NP) ink is a colloidal dispersion system typically formed by uniformly dispersing NiO_x_ NPs in an aqueous solution. Leveraging the quantum size effect and high surface energy of NPs, the ink containing these NPs could be used to prepare the thin films that serve as the efficient HTLs in PSCs; that is, the fabrication of high‐quality NiO_x_ HTLs with efficient hole extraction and excellent transport could be obtained via facile solution processing [74]. The mechanism of this method is that the NiO_x_ NPs formed during the synthesis process could generate a suitable amount of Ni vacancies (*V*_Ni_), thereby forming enough holes within the materials. Thus, the obtained NiO_x_ NPs exhibit *p*‐type semiconducting transport characteristics, which determine the electrical conductivity and hole transport performance of the thin films [64, 75, 76]. In addition, the stability and uniformity of NPs within the dispersion system also directly influence the quality of the synthesized thin films [67, 77]. Current research has demonstrated that the NiO_x_ thin films fabricated using the pre‐synthesized NP method typically exhibit high crystalline and film quality and low trap density. Furthermore, this method shows excellent compatibility with large‐area processing and flexible substrates, coupled with its cost‐efficiency feature, making it highly suitable for industrial‐scale production [16, 78, 79]. Beyond the related functions, the obtained NiO_x_ thin films through this pre‐synthesized NP process could also ensure stable perovskite photovoltaic device performance through mature processes, reduce the costs in large‐scale production, and feature convenient supply chains. Such a method would provide reliable support for the research, development, and industrialization of perovskite photovoltaics; currently, the devices based on commercial NiO_x_ NPs have achieved excellent device performance, with the PCE exceeding 27% [3, 5, 80, 81].

**FIGURE 4:**
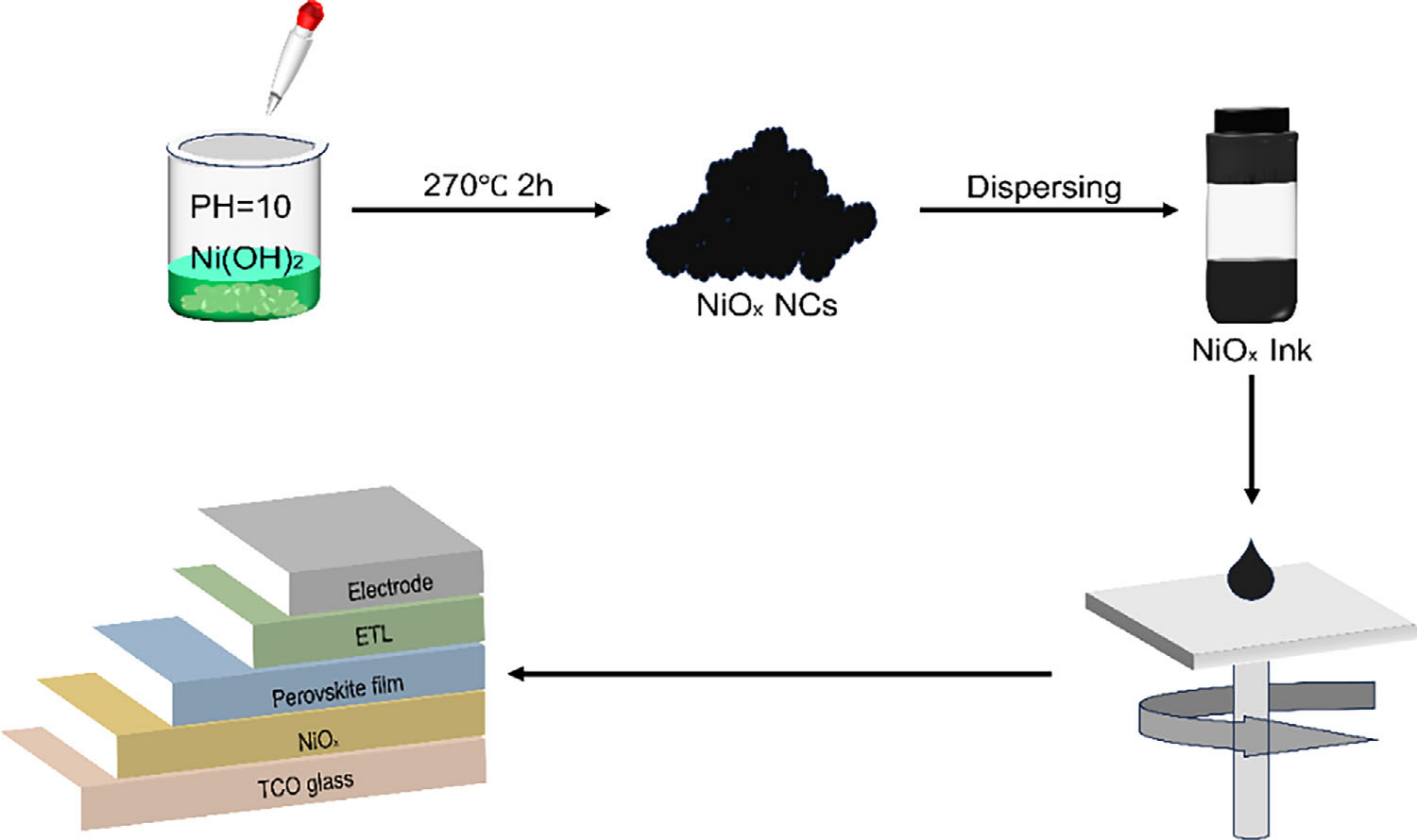
Schematic diagram of the synthesis of NiO_x_ NPs, the preparation of NiO_x_ thin film, and fabrication of the related perovskite photovoltaic device.

Figure 4 clearly illustrates the process flow of the pre‐synthesized NP method for synthesizing NiO_x_ thin films. The related reaction mechanisms can be explained in Equations (1) and (2). This method initially involves the reaction of nickel salt precursors, such as nickel nitrate, with strong bases (e.g., NaOH) to yield nickel hydroxide Ni(OH)_2_ precipitates (Equation 1) [67, 77, 79]. Subsequently, this precipitate is calcined at about 270°C to form NiO_x_ NPs (Equation 2). Thereafter, the obtained NPs are dispersed in a solvent (such as water or alcohol) to form a stable colloidal dispersion system (that is, the NiO_x_ ink) [82]. Finally, the related NiO_x_ thin films can be obtained through solution‐based processes such as spin‐coating and spray‐coating, which do not require any additional high‐temperature annealing steps [79].

(1) $$\mathrm{Ni}{\left(NO_{3}\right)}_{2}+\;\mathrm{NaOH}\;\to \;\mathrm{Ni}{\left(\mathrm{OH}\right)}_{2}↓+\;\mathrm{NaN}O_{3}$$

(2) $$\mathrm{Ni}{\left(\mathrm{OH}\right)}_{2}\to \;\mathrm{Ni}O_{X}+H_{2}O$$

Based on the above discussions, such a straightforward, easily operated method for synthesizing NiO_x_ HTLs would hold great promise for cost‐effectiveness, high‐efficiency photovoltaic cells for practical applications.

#### 4.1.2 Existing Issues

Notably, despite the obvious advantage of the method and its great potential, the preparation of high‐performance NiO_x_ HTLs using the pre‐synthesized NiO_x_ NP method to meet the requirements of high‐efficiency and stable PSCs still faces several scientific challenges and technical bottlenecks. First, NiO_x_ NPs’ dispersibility in the solvent is highly pH‐dependent, which would influence the quality of NiO_x_ NPs and the obtained thin films, thus affecting the related photovoltaic device performance directly [67, 71, 77]. Also, the dispersion stability of NiO_x_ NPs is insufficient. In detail, NiO_x_ NPs synthesized via the chemical precipitation method tend to adsorb impurity ions such as NO^3−^ and OH^−^, and these ions are highly pH‐sensitive. Deviating from the optimal pH range causes agglomeration or uneven dispersion, impairing the HTL uniformity and thus the photovoltaic device reproducibility [67, 77, 78].

Second, pure NiO_x_ NPs normally have relatively low hole concentration, due to the high ionization energy of *V*_Ni_, limiting their electrical conductivity and carrier transport. In addition, although the doping method could mitigate this issue, the excessive doping (e.g., with alkali or transition metals) could induce lattice distortion, reduce transparency, and increase defect states [68, 83]. Furthermore, highly active surface Ni^3+^/Ni^4+^ species on NiO_x_ thin films tend to undergo redox reactions with I^−^ in perovskite active layers, causing interfacial degradation between NiO_x_ layers and perovskite active layers [84].

In addition to the above factors, the surface defects (such as pinholes and dangling bonds) in NiO_x_ films made by the NP inks could exacerbate non‐radiative recombination, thus reducing the open‐circuit voltage (*V*_OC_) and *FF* values of the fabricated perovskite photovoltaic cells, which is particularly a prominent phenomenon in the large‐area fabrication of solar cell devices. Furthermore, the rheological properties of solution‐processed inks are difficult to precisely regulate, which easily leads to the non‐uniform thickness of NiO_x_ thin films [85, 86]. Therefore, even though this method is capable of low‐temperature thin‐film formation and compatible with both flexible and large‐scale device fabrications, there are still some issues that should be well addressed without any treatments, which affect long‐term stability and performance of the relevant photovoltaic devices, thus collectively limiting their further commercial applications [21].

#### 4.1.3 Strategies for Relevant PSC Performance Optimization

##### 4.1.3.1 Doping: Achieving Coordinated Control of Energy Levels, Carrier Transport, and Interface Properties

So far, there have been some promising strategies developed to address the above‐discussed challenges of NiO_x_ HTLs through the pre‐synthesized NP method. Notably, metal ion doping, due to its convenience and versatility, has become a widely used strategy for optimizing the quality of functional materials. In this regard, doping processes have also been used in this method, aiming to improve the quality of NiO_x_ NP inks and the related thin films, as well as their physical properties, thus beneficial for improving the photovoltaic device performance. From a mechanistic perspective, the core of doping lies in systematically adjusting the energy band structure, carrier concentration, and mobility of NiO_x_ by introducing external ions, and influencing its surface chemical state, thereby optimizing hole extraction and transmission.

**FIGURE 5:**
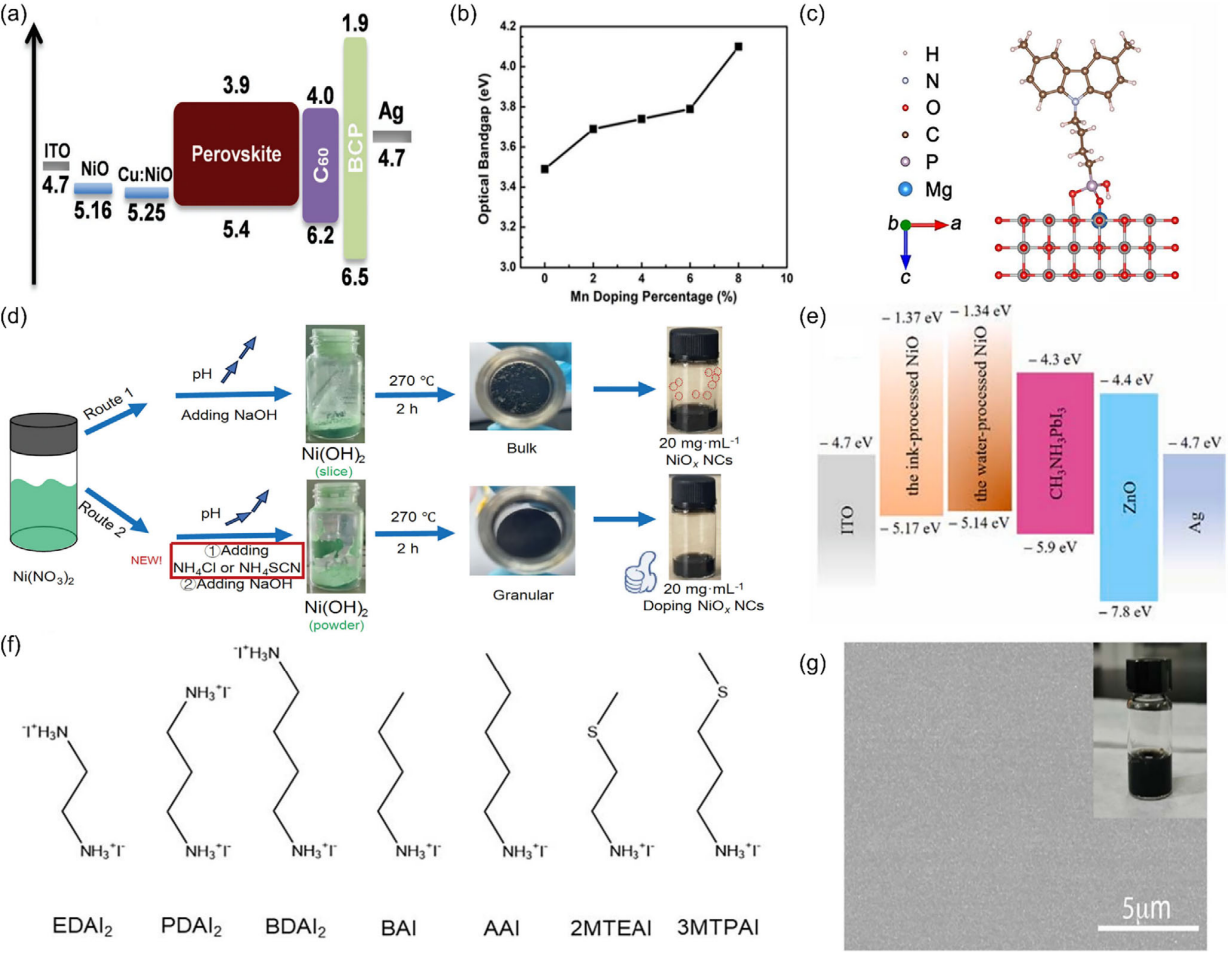
(a) Schematic diagram of energy levels in the inverted PSCs with Cu:NiO_x_ working as HTL. Reproduced with permission [71]. Copyright 2018, Wiley‐VCH. (b) Variation of optical bandgap values of NiO_x_ NPs with different Mn doping concentrations. Reproduced with permission [87]. Copyright 2025, Royal Society of Chemistry. (c) Model diagrams of Me‐4PACz deposited respectively on NiO_x_. Reproduced with permission [88]. Copyright 2025, Wiley‐VCH. (d) Synthesis method for both the undoping and doping NiO_x_ NCs. Reproduced with permission [89]. Copyright 2024, Youke Publishing Co. (e) Energy band alignment of the ITO/NiO_x_/CH_3_NH_3_PbI_3_/ZnO/Ag solar cell. Reproduced with permission [90]. Copyright 2024, Elsevier. (f) Chemical structures of the diammonium and ammonium ligands investigated in this study. Reproduced with permission [91]. Copyright 2023, The American Association for the Advancement of Science. (g) SEM image of a typical NiO_x_ thin film prepared from the NiO_x_ NPs ink ([NH_3_]/[Ni] = 3). Reproduced with permission [55]. Copyright 2021, IOP Publishing Ltd.

In 2018, Chen et al. successfully synthesized copper‐doped NiO (Cu:NiO) NP ink and also prepared the related HTLs via a room‐temperature spin‐coating method [71]. The obtained thin films show high quality even without any high‐temperature annealing treatments. Specifically, the Cu doping was achieved by adding the 5% copper (II) nitrate trihydrate into the nickel nitrate precursor. As shown in Figure 5a, Cu doping not only induces an upward shift of the acceptor energy level toward the VBM but also, through the Cu^+^/Cu^2+^ co‐doping, replaces Ni^2+^ sites and significantly increases the carrier concentration of HTLs (from 5.3 × 10^18^ cm^−3^ to 7.3 × 10^19^ cm^−3^) and their carrier mobility (from 0.12 cm^2^ V^−1^ s^−1^ to 2.53 cm^2^ V^−1^ s^−1^). Based on this optimization process, the small‐area photovoltaic device (0.08 cm^2^) could achieve a PCE of over 20%; significantly, the large‐area device (1 cm^2^) exhibits a high PCE of 18.07%, with the flexible one reaching a PCE of 17.41%. Furthermore, after being stored under 50%–65% relative humidity for 1000 h, the devices still retained 95% of their initial efficiency. This work showed that such a simple doping process (even without an annealing step) would be an effective strategy for significantly optimizing the NiO_x_‐based PSC device performance and realizing the large‐scale and flexible functions, highlighting its potential in real‐world photovoltaic device applications. Moreover, this study also revealed the physical mechanism; that is, metal ion doping of HTLs could enhance photovoltaic device performance through energy level regulation and optimization of carrier transport in the device, laying an important foundation for further designs of doping strategies.

With the deepening of research on metal ion doping, the potential of other metal ions, like transition metal doping, in the synergistic regulation of NiO_x_ microstructure and optoelectronic properties has attracted increasing attention. In 2025, Islam et al. successfully prepared Mn‐doped NiO_x_ NPs via a chemical precipitation method [87]. The study showed that, as the Mn doping concentration increased, the grain size of NPs would decrease from 15.26 nm to 10.38 nm, and their band gap could expand from 3.49 eV to 4.10 eV (Figure 5b). With a 6‐wt% Mn doping, the mobility of NiO_x_ NPs could reach 1.31 × 10^3^ cm^2^ V^−1^ s^−1^ (2.11 × 10^2^ cm^2^ V^−1^ s^−1^ for pure NiO_x_ NPs). Combined with SCAPS‐1D simulations, the potential of NiO_x_ NPs working as an effective HTL was verified, indicating that transition metal doping could also achieve synergistic optimization of optical and electrical properties of NiO_x_ NPs through the structural regulation. This work expanded the research scope of transition metal doping systems and revealed the intrinsic correlation between grain size and band gap variation through systematic structural characterization. However, it should be noted that the simulation prediction results still require systematic experimental verification through actual device fabrication and testing. In 2025, Zhang et al. introduced Mg^+^ doping when synthesizing NiO_x_ NPs [88]. Mg‐doping increases the hydroxyl density on the surface of NiO_x_ and provides denser binding sites for the Me‐4PACz (Figure 5c), forming denser and more uniform HTLs. This effectively passivates the bottom‐buried interface defects of the perovskite active layer and optimizes its crystallization environment. In terms of electrical properties, Mg:NiO_x_ exhibits higher electrical conductivity and better energy level alignment, promoting efficient hole extraction and transport. Ultimately, the champion device using Mg:NiO_x_ HTL achieved a photoelectric conversion efficiency of up to 25.86%. This work demonstrates that doping not only regulates the electrical properties of the bulk phase, but also affects interface passivation and perovskite crystallization by altering the surface chemistry, demonstrating a multi‐dimensional regulatory effect.

Against the backdrop where metal ion doping has primarily focused on improving the carrier concentration of NiO_x_ NPs, innovations in doping strategies continued to drive photovoltaic performance breakthroughs. Non‐metal ion doping of NiO_x_ NPs has also made significant progress due to its unique advantages in impurity suppression and precise valence state regulation. Compared with the lattice distortion issues that may be caused by metal ion doping, non‐metal ion (like ammonium salt) doping focuses more on impurity suppression and valence state regulation, providing an effective pathway for precise energy level matching. Song et al. synthesized NiO_x_ NPs with the doping of ammonium salts (NH_4_Cl/NH_4_SCN) (Figure 5d) [89]. Through the pH regulation, the growth of Ni(OH)_2_ precipitates was effectively inhibited, thus the nitrate (NO^3−^) impurities being reduced and simultaneously the Ni^3+^/Ni^2+^ ratio being increased from 0.848 to 1.775 [78]. This strategy of using non‐metal ion doping could also optimize the energy level alignment of NiO_x_ thin films, thus reducing their VBM offset to 0.03 eV. When combining this strategy with using the MeO‐2PACz ((2‐(3,6‐Dimethoxy‐9H‐carbazol‐9‐yl)ethyl)phosphonic acid) SAM as interface passivation, the relevant photovoltaic device could exhibit a high PCE of up to 23.27%. This work showed the synergistic effect of both the energy level matching and the interface passivation of the photovoltaic device performance; however, it should be noted that the influence of ammonium salt doping concentration on the uniformity of NiO_x_ films would still require further investigations and optimizations.

Based on the above, the studies adopted the doping strategy via using different dopants, ranging from Cu ions, which realized the simplification of preparation processes, to Mn and Mg ions, which explored the correlation between structure and performance, and further to ammonium salt ions that achieved multi‐dimensional regulation for improving PSC performance and stability. These clearly reflect the shift of research from single‐dimensional conductivity enhancement to multi‐dimensional and systematic performance regulation and enhancement, providing diversified optimization pathways for solution‐processed NiO_x_ HTLs for the practical applications of the relevant PSC devices. These studies collectively reveal the core mechanism of the doping strategy: through ion substitution and valence state regulation, a systematic optimization of the band structure, carrier dynamics, and interface properties is achieved, providing universal design principles for high‐performance hole transport layers.

##### 4.1.3.2 Annealing and Process Optimization: Reducing Defect Density and Improving Interface Contact

In addition to intrinsic modification through chemical doping, optimizing the post‐processing techniques for film preparation is another key strategy. The core mechanism lies in controlling the film formation conditions to reduce the defect density of the NiO_x_ layer itself and improve the interface contact quality between it and the substrate, as well as the perovskite layer. Doping engineering has significantly enhanced the quality and physical properties of NiO_x_ HTLs and thus the relevant photovoltaic device performance. Traditional high‐temperature preparation techniques for large‐scale PSCs not only consume high energy but also struggle to adapt to flexible polymer substrates. In addition, the extensive use of organic solvents would introduce environmental risks and cost pressures. To tackle these issues, Chen et al. synthesized the aqueous NiO_x_ NP inks in 2024, which could form mesoporous films after the treatment at 100°C [90]. This approach is highly compatible with flexible substrates (such as PET and PEN); the relevant MAPbI_3_‐based flexible solar cells with the NiO_x_ HTLs via this process could achieve a high PCE of 11.24%, comparable to the performance of the device with NiO_x_ HTLs prepared via high‐temperature processes. In addition, this method could replace organic solvents with an aqueous system while significantly reducing the after‐processing temperature, which not only lowers the fabrication energy consumption but also avoids the pollution risks. Based on the above, this method provides a feasible solution for the large‐scale fabrication of environmentally friendly, high‐performance perovskite photovoltaic devices. However, the energy level alignment between the VB of aqueous NiO_x_ and that of the perovskite layer is increased (Figure 5e), and its efficiency still lags the current state‐of‐the‐art levels. Therefore, how to further improve the crystallinity and electrical conductivity of aqueous NiO_x_ HTLs under relatively low‐temperature conditions remains an issue to be addressed in future research. This work demonstrates that the core advantage of the low‐temperature annealing process lies in reducing the damage to the flexible substrate caused by high temperatures and lowering energy consumption. However, the challenge is how to simultaneously achieve low defect density, high crystallinity, and good energy level matching of the thin film under these conditions.

##### 4.1.3.3 Multi‐Strategy Integration: Collaborative Defect Management and Performance Improvement from Bulk Phase to Interface

As the research progresses, a single modification strategy is often insufficient to solve all interface problems. Therefore, by integrating multiple approaches and conducting collaborative engineering on the phase, surface, and interface of NiO_x_ with perovskite, it becomes an important way to achieve efficient and stable devices. The physical and chemical mechanisms focus on the comprehensive passivation of defects, the optimization of the carrier transport interface, and the regulation of the perovskite crystallization environment. Beyond the relatively low temperature synthesis route, the integration of basic research and application exploration is also improving the research system of NiO_x_ NP inks. Zhang et al. found that the undoped NiO_x_ NPs, working as HTLs, could achieve efficient hole transport by optimizing the NP size and dispersibility, highlighting the potential of pure‐phase NiO_x_ as suitable HTLs in low‐cost and high‐performance PSC applications [92]. Pious et al. optimized wide‐bandgap perovskite photovoltaic devices through interface engineering and found that the uniformity of NiO_x_ NP ink is a key factor in enhancing the performance of large‐area photovoltaic devices [93]. Moreover, the grain sizes of the synthesized NiO_x_ thin films became more uniform, resulting in the fewer pinholes within the films. When combined with (2‐(9H‐carbazol‐9‐yl)ethyl)phosphonic acid (2PACz), the obtained NiO_x_ thin films could show the optimized potential distribution and demonstrate the improved carrier transport capability, thus resulting in an improvement in short‐circuit current density (*J*_sc_) in the relevant photovoltaic devices. This demonstrates a synergistic mechanism where the properties of the nanoparticles themselves (such as size and dispersion) are optimized to reduce the bulk defects in the film, and the interface molecular layer is combined to improve the contact. Furthermore, Liu et al. proposed a bimolecular passivation strategy; that is, Sulfur‐modified methylthiol molecules (such as 3MTPAI) are used to achieve chemical passivation of NiO_x_ thin films by forming strong coordination and hydrogen bonds with surface defects of perovskites [91]. Meanwhile, diammonium molecules (such as PDAI_2_) are introduced to repel minority carriers through field‐effect passivation (Figure 5f), thus reducing contact‐induced interfacial recombination. This method could increase the carrier lifetime of NiO_x_ HTLs by 5 times and reduce the loss of photoluminescence quantum yield (PLQY) by 1/3, thus enabling the photovoltaic devices to achieve a certified quasi‐steady‐state efficiency of 25.1% and allowing them to operate stably for over 2000 h in an ambient atmosphere at 65°C. Additionally, all‐perovskite tandem solar cells with an efficiency of 28.1% were successfully obtained, verifying the versatile regulatory potential of such the pre‐synthesized NP/NC method in interface engineering and device performance optimizations. This strategy clearly demonstrates the synergistic effect of two different mechanisms, namely chemical passivation and field‐effect passivation, in eliminating interface defects and inhibiting non‐radiative recombination.

Despite numerous improvement methods, the requirements such as low‐pH‐value environment remain the problem being addressed. Mild synthesis conditions can provide a basis for future industrialization and practical application. To tackle this issue, Tong et al. adjusted the proportion of ammonia and found that NPs with good dispersibility and crystallinity, as shown in Figure 5g, could be obtained within a broader range of pH values [55]. This work is carried out from the perspective of synthetic chemistry. By adjusting the precursor environment, high‐quality nanocrystals are obtained, thereby reducing the defects introduced during the synthesis process at the source.

**FIGURE 6:**
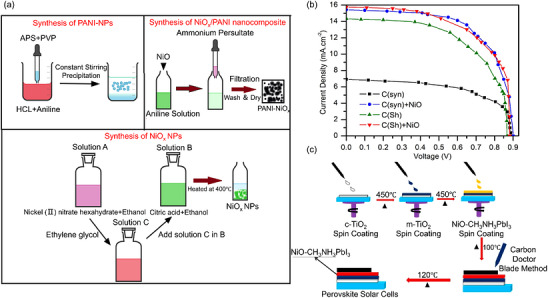
(a) Synthesis of PANI‐NPs, NiO_x_/PANI nanocomposite and NiO_x_ NPs. Reproduced with permission [94]. Copyright 2024, Royal Society of Chemistry. (b) *J–V* curve of the best PSCs with synthesized (syn) and commercial (sh) carbon cathode doped with NiO NPs. Reproduced with permission [95]. Copyright 2024, Elsevier. (**c**) Fabrication route of the relevant PSCs. Reproduced with permission [42]. Copyright 2024, Elsevier.

Except for the core functionality as the HTLs, the innovative use of NiO_x_ NPs in carbon‐electrode‐based PSCs has further expanded their functional boundaries. Carbon‐electrode‐based PSC devices have garnered widespread attention due to the advantages of low cost and high stability offered by carbon electrodes; however, the issues such as the insufficient intrinsic conductivity of carbon materials and poor interfacial contact have hindered device performance improvement [96]. Kumar et al. designed a carbon paste doped with NiO_x_/polyaniline (PANI) nanocomposites (Figure 6a) [94]. Through stepwise energy level alignment and enhanced interfacial contact, the PCE of the photovoltaic device could improve from 14.46% to 18.30%, and it could retain 96% of its initial efficiency after 2400 h. This demonstrates the unique advantage of inorganic‐organic composite systems in balancing conductivity and stability. Similarly, Nakhaeenejad et al. synthesized NiO_x_ NPs and mixed them with carbon paste and then performed annealing treatment at 450°C to form composite electrodes [95]. When the composite ratio of NiO_x_ NPs was 1:30 (mass ratio), the pores generated after high‐temperature treatment enabled tighter contact between the carbon electrode and the perovskite layer, significantly optimizing charge transport performance and enhancing the *V*_OC_. As shown in Figure 6b, the device PCE increased from 3.3% to 8.6%. The underlying mechanism is that NiO_x_ NPs capture electrons through surface defect states, suppressing the non‐radiative recombination between the carbon electrode and the perovskite layer. These two studies jointly confirm that NiO_x_ NPs can not only function as HTLs but also remedy the defects of carbon materials through electrode modification, providing a new direction for the design of low‐cost, high‐performance devices. In line with this, Arjun et al. synthesized NiO_x_ NPs and directly incorporated them into CH_3_NH_3_PbI_3_ active layers (Figure 6c) [42]; the related champion device achieved a high *J*_sc_ of 22.79 mA/cm^2^ and a high PCE of 13.43%. After a 600‐h stability test, the device retained 93% of its efficiency, confirming that NiO_x_ NPs can not only serve as HTLs but also optimize charge transport by modifying the quality of the perovskite layer. This study complements electrode modification strategies, expanding the application scenarios of NiO_x_ NPs from the perspective of active layer optimization and highlighting the diversity of their functions. This series of studies has revealed that NiO_x_ NPs, as a multifunctional additive, can exert its effects by extending from the hole transport layer to electrode modification and even the optimization of the perovskite active layer. This is achieved by introducing defect passivation centers at different positions or improving the contact to enhance the overall device performance.

##### 4.1.3.4 Surface and Interface Molecular Engineering: Interface Composites Inhibition and Transmission Optimization

**FIGURE 7:**
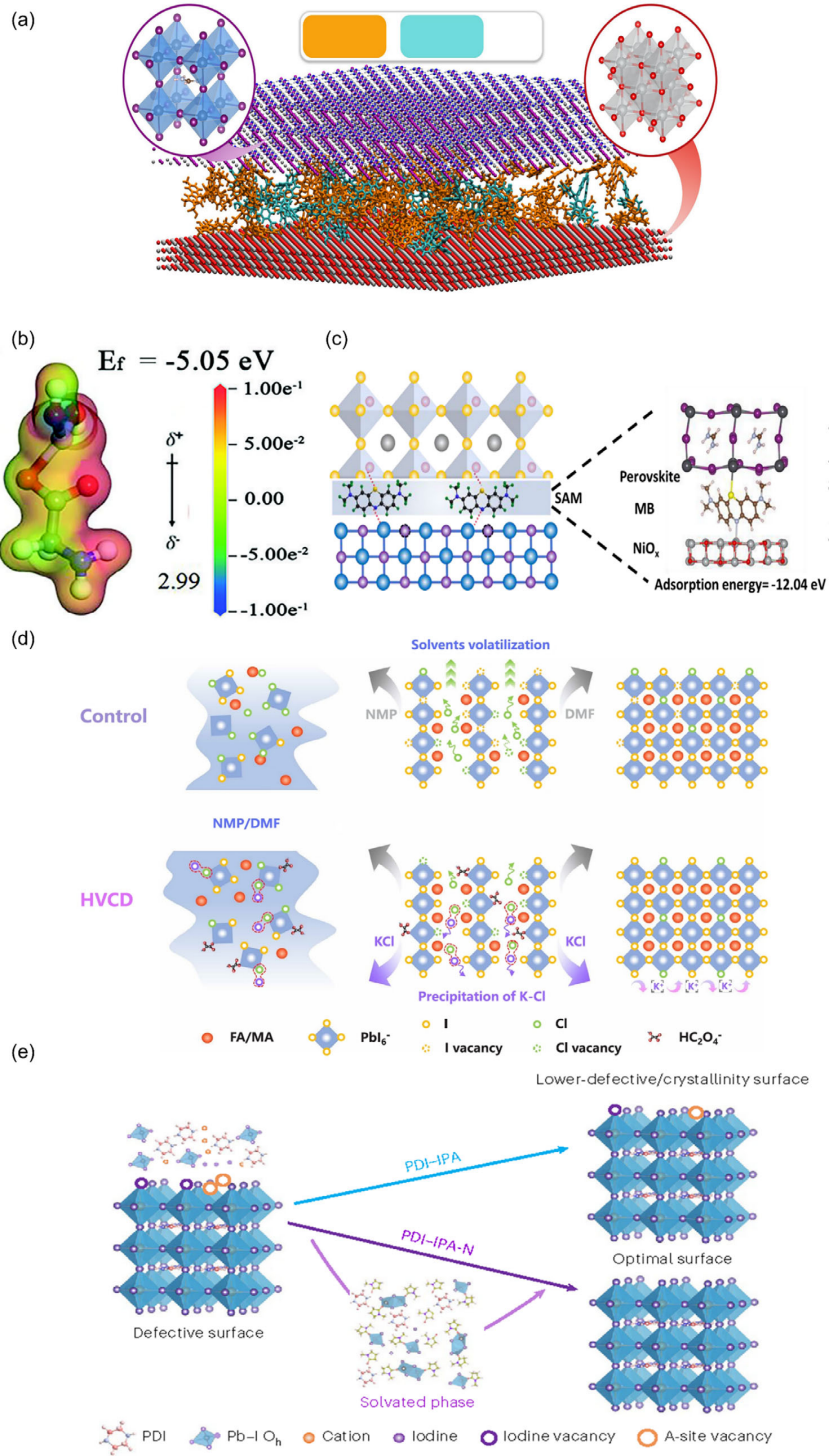
(a) Illustration of the NiO/SAMs/perovskite heterojunction model for molecular dynamics simulations. Reproduced with permission [97]. Copyright 2024, Springer Nature Limited. (b) The ESP images of AG molecule. Reproduced with permission [81]. Copyright 2025, Wiley‐VCH. (c) DFT calculation of MB in the interface. Reproduced with permission [98]. Copyright 2025, Royal Society of Chemistry. (d) Schematic diagram of the redistribution of Cl anions in the HVCD strategy. Reproduced with permission [5]. Copyright 2025, The American Association for the Advancement of Science. (e) Schematic illustration of the mechanism of the IPA‐N treatment, showing phase conversion via the solvated intermediate phase on perovskite surfaces. Reproduced with permission [3]. Copyright 2025, Springer Nature Limited.

For devices based on commercialized NiO_x_ nanoparticles, surface and interface engineering are the key factors determining their performance limit. The core concept of these strategies is to achieve atomic‐level precision control over the surface energy levels, defect states, chemical reactivity, and perovskite nucleation interfaces of the NiO_x_ surface by introducing molecular monolayers or additives. Beyond the recent lab‐based work on the synthesis of NiO_x_ nanocrystals, commercially available NiO_x_ NPs are widely used due to their mature manufacturing processes and stable properties. Recently, Liu et al. formulated commercial NiO_x_ NPs into inks and employed them as HTLs [97]. Using a molecular hybridization strategy, the authors co‐assembled the widely used self‐assembled molecule Me‐4PACz (4‐(3‐(3‐methyl‐6‐phenylcarbazol‐9‐yl)propyl)benzaldehyde) (to optimize the interface level and facilitate the extraction of holes) with 4,4',4''‐nitrilotribenzoic acid (NA) to form NA‐Me mixed SAMs, aiming of modifying the surface of NiO_x_ thin films and their physical properties (Figure 7a). This design aims to address two key interface issues. The first is the problem that the Me‐4PACz molecule tends to aggregate on the surface of NiO_x_, which leads to uneven coverage and disorder in energy levels; the second is the adverse effect of surface defects (such as NiO‐OH) on the precursor of the perovskite. The introduction of NA not only inhibits the aggregation of Me‐4PACz through intermolecular interactions, but also improves the uniformity and coverage of the SAM layer. Its carboxyl functional groups may also interact with the surface sites of NiO_x_, optimizing the interface wettability and partially passivating the surface defects. The mechanism of this strategy lies in the molecular co‐assembly, which simultaneously optimizes the physical coverage and chemical uniformity of the SAM layer, and cooperatively achieves effective regulation of the surface energy levels (through Me‐4PACz) and improvement of the interface morphology/stress (through NA), thereby synchronously enhancing the carrier extraction efficiency and suppressing non‐radiative recombination caused by interface defects and stress concentration. Based on this strategy, the *p‐i‐n* inverted PSCs achieved the certified steady‐state efficiencies of 26.54% for the small‐area devices and 22.74% for the larger‐area (11.1 cm^2^) mini‐modules. Notably, the devices retained 96.1% of their initial efficiency after 2400 h of 1‐sun illumination in ambient air, demonstrating excellent scalability and stability for large‐area device fabrications. The research groups led by Cheng and Qu also utilized self‐assembled molecules to boost PSC device performance (Figure 7b,c), achieving the high PCE values of 26.74% (certified 26.21%)and 26.39%, respectively [81, 98]. Both worked on the Me‐4PACz SAMs (used for optimizing energy level matching) and introduced functional molecules to construct composite systems to improve the performance of NiO_x_ HTLs. Cheng et al. combined Me‐4PACz with aluminum glycinate (AG). The Al‐OH group of AG could bind to the unanchored O═P─OH and exposed NiO‐OH defects on the surface of NiO_x_ to achieve passivation. Meanwhile, the inhibition of Me‐4PACz agglomeration reduced the surface roughness of NiO_x_ layer from 10.14 nm to 7.09 nm, and the energy level matching with the perovskite layer was optimized by down‐regulating the Fermi level of Me‐4PACz (from −4.65 eV to −4.98 eV). Qu et al. co‐adsorbed Me‐4PACz with methylene blue (MB). MB improved the dispersion of Me‐4PACz on the NiO_x_ matrix surface through π‐π interaction, reducing voids and agglomeration to form a smooth and dense interface layer. Meanwhile, as a redox mediator, it selectively reduces the Ni^4+^ on the surface of the NiO_x_ layer to inhibit interfacial side reactions and increases the surface conductivity of the NiO_x_ layer (the average surface current increases from 3.53 nA to 6.81 nA), accelerating hole extraction.

Furthermore, Xiong et al. introduced H_2_O_2_ as an additive into the NiO_x_ aqueous solution during the preparation of NiO_x_ HTL [80]. This approach was proven to effectively enhance the dispersion stability of NiO_x_ nanoparticles, significantly mitigate aggregation, and optimize the chemical state of nickel by regulating the Ni^3+^ ratio, thereby ensuring ideal electrical conductivity and facilitating efficient hole transport. Building on this prior foundation, the same group subsequently combined this optimized HTL with a homogenized vertical chlorine distribution (HVCD) strategy for optimizing the perovskite active layer (Figure 7d). This integrated approach ultimately resulted in the fabricated photovoltaic devices achieving a remarkable steady‐state certified PCE of 27.2% [5]. Similarly, in the improvement of perovskite crystallization performance, Liu et al. recently reported a solvated intermediate‐driven surface transformation strategy [3]. By introducing trace amounts of *N*‐Methyl Pyrrolidone (NMP) into the IPA solution of piperazinium diiodide (PDI), high‐quality *α*‐phase perovskite is directly formed from the solvated intermediate phase, bypassing the *δ*‐intermediate phase that is prone to defects (such as V_I_ and V_Pb_) (Figure 7e). The certified PCE of the PSC fabricated using the HTL prepared with NiO_x_ NPs and the SAM prepared with Me‐4PACz and NA (used for optimizing energy level matching) reached up to 27.27%. After continuous operation at 65°C and 1‐sun illumination for 2,500 h, the device still maintained an initial efficiency of 96%. Meanwhile, some other studies have achieved a high PCE of over 24%, which fully demonstrates the high applicability of commercial NiO_x_ NPs in practical PSC applications [19, 80, 99].

**FIGURE 8:**
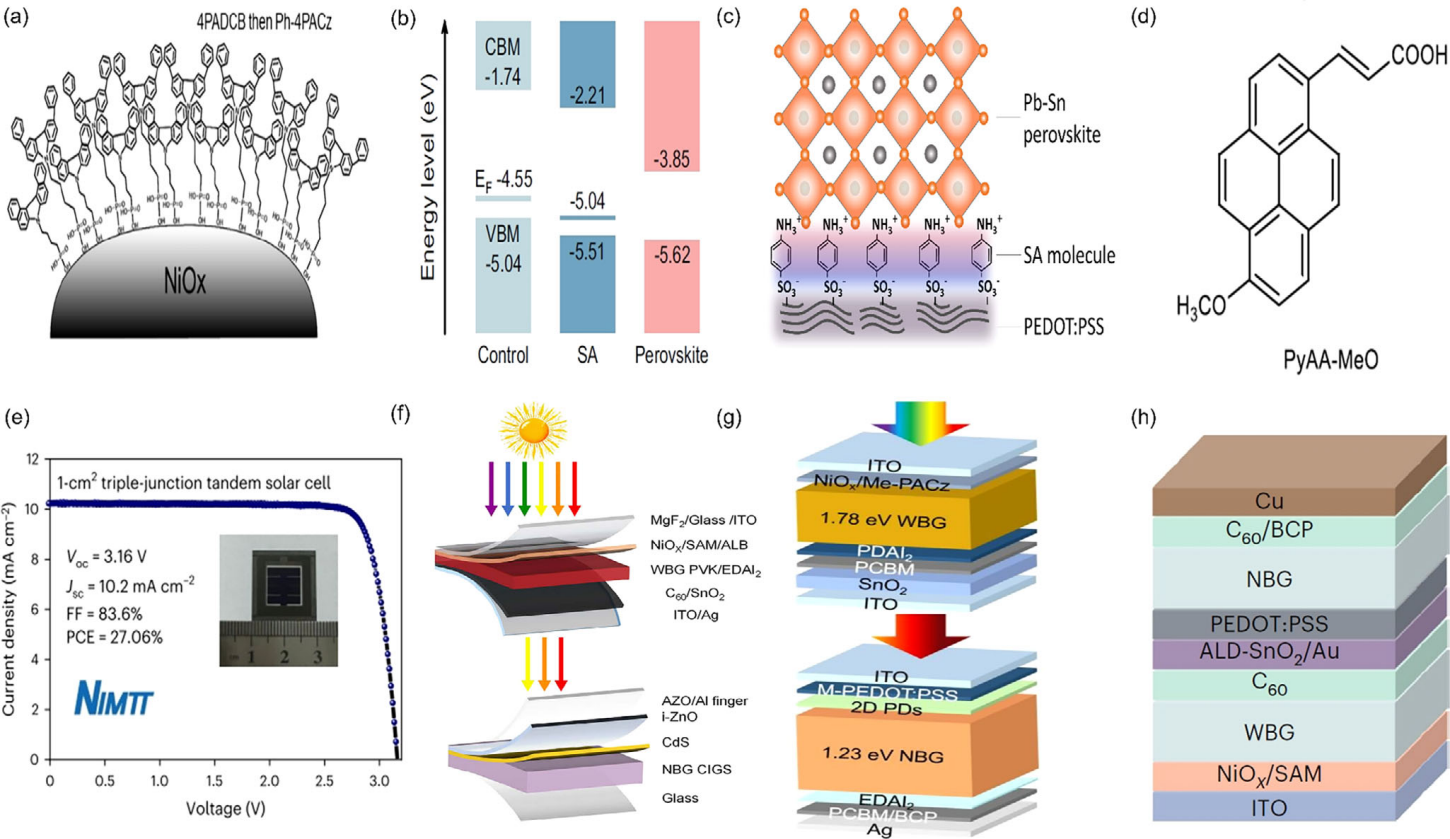
(a) Using sequential deposition, 4PADCB is deposited first and then Ph‐4PACz, forming a compact and ordered molecular arrangement, reducing voids and improving interface uniformity. Reproduced with permission [100]. Copyright 2025, American Chemical Society. (b) Energy levels of VBM and CBM derived from UPS spectra of the control and SA‐treated substrates. Reproduced with permission [101]. Copyright 2025, Springer Nature Limited. (c) Schematic illustration of dipolar passivation at the HTL/mixed Pb‐Sn perovskite interface. Reproduced with permission [4]. Copyright 2025, Springer Nature Limited. (d) Chemical structures of PyAA‐MeO. Reproduced with permission [102]. Copyright 2025, Springer Nature Limited. (e) Third‐party‐measured reverse‐scan *J*–*V* curves of champion triple junctions: 1 cm^2^. Reproduced with permission [103]. Copyright 2025, Springer Nature Limited. (f) Schematic of the 4T PVK/CIGS TSCs. Reproduced with permission [104]. Copyright 2025, Wiley‐VCH. (g) Illustration of the device structure of the 4T all‐perovskite tandem solar cells. Reproduced with permission [105]. Copyright 2025, Royal Society of Chemistry. (h) Device architecture of 2T all‐perovskite TSCs. Reproduced with permission [106]. Copyright 2025, Springer Nature Limited.

In addition, NiO_x_ NPs have also shown great application potential as efficient HTL in high‐efficiency multi‐junction solar cells such as perovskite/silicon tandem cells and all‐perovskite tandem cells. In the realm of interface engineering and energy level optimization, research focuses on forming denser molecular layers and optimizing energy level alignment. Recently, He et al. reported an innovative sequential deposition method [100], and they first deposited (4‐(7H‐diphenyl‐[c,g] carbazol‐7‐yl) butyl)phosphonic acid (4PADCB) on the surface of NiO_x_, and then deposited [4‐(3,6‐diphenyl‐9H‐carbazol‐9‐yl) butyl]phosphonic acid (Ph‐4PACz) (Figure 8a). The core advantage of this strategy lies in the fact that the latter can effectively fill any potential vacancies in the former's molecular layer, thereby forming a more dense and uniform supramolecular structure on the surface of NiO_x_ compared to traditional single or mixed self‐assembled films, and inhibiting Ni^≥4+^ defects (such as NiO‐OH). XPS analysis indicated that the proportion of Ni^3+^ conducive to hole transport increased from 54% to 61%, and the VB offset between NiO_x_ and the perovskite layer was significantly reduced from 0.54 eV to 0.07 eV, which greatly promoted hole extraction and suppressed recombination. Ultimately, the single‐junction perovskite cell based on this optimized NiO_x_ interface achieved a high *V*_OC_ of 1.33 V and a high PCE of 20.35% and further contributed to the all‐perovskite tandem cell achieving a champion PCE of 27.03%. In the same year, Shi et al. introduced 6‐aminohexane‐1‐sulfonic acid (SA) as a co‐adsorbate to form a mixed SAM with Me‐4PACz, effectively optimizing the coverage density and energy level arrangement of the NiO_x_ HTL (Figure 8b). The more uniform and denser SAM layer brings two benefits. Firstly, it achieves more consistent control of the surface energy level, improving the arrangement of energy levels. Secondly, it reduces the exposed NiO_x_ surface defect sites (Ni^≥4+^) due to the uneven SAM coverage, thereby directly reducing the interface non‐radiative recombination losses caused by these defects. Thus, significantly improving the performance of single‐junction devices (with PCE of 20.67% and certified 20.21%) and all‐perovskite tandem cells (with PCE of 28.94% and certified 28.78%) [101]. Meanwhile, Lin et al. developed a dipolar passivating strategy using sulfanilic acid (SA), whose ‐NH_3_^+^ ends anchor the perovskite surface to passivate the deep‐level trap defects including iodine vacancies (V_I_) and undercoordinated Pb^2+^/Sn^2+^ sites, while its ‐SO_3_^−^ groups orient toward the HTLs to form a specifically oriented dipolar layer (Figure 8c), which thus effectively reduces interfacial non‐radiative recombination, optimizes energy level alignment and enhances carrier extraction efficiency [4]. Ultimately, the PCE of the resulting all‐perovskite tandem devices, with NiO_x_ (modified by MeO‐2PACz and 2PACz to optimize the interface level and facilitate the extraction of holes) thin films working as HTLs, was increased to 30.6% (along with a certified steady‐state PCE of 30.1%), setting a record in this field. This work significantly drove the all‐perovskite tandem devices. Furthermore, Luo et al. addressed the key issue of energy level mismatch in the HTL in wide‐bandgap perovskite cells [102]. They designed a series of SAM molecules based on fully conjugated pyrene nuclei, among which (E)−3‐(6‐methoxypyren‐1‐yl)acrylic acid (PyAA‐MeO) (Figure 8d), with the strongest electron‐donating ability targets and mitigates interfacial defects of wide‐bandgap perovskites (including deep‐level traps, undercoordinated Pb^2+^ sites, and non‐radiative recombination induced by charge accumulation), forming the most favorable energy level alignment with wide‐bandgap perovskites and providing the largest intrinsic driving force for hole extraction. This work introduces NiO_x_ bonding layers in tandem devices to optimize device performance. The single‐junction wide‐bandgap perovskite cell based on PyAA‐MeO‐modified NiO_x_ HTL achieved a PCE of 22.8% and, when combining with the crystalline silicon TOPCon bottom cell, the tandem device showed a certified PCE as high as 30.9% (laboratory efficiency 31.1%). Most recently, Zheng et al. replaced the unstable LiF (between the perovskite and the C_60_ layer) in the top wide‐bandgap perovskite junction with piperazine‐1,4‐diium chloride (PDCl), which specifically targets and passivates critical defects including surface PbI_2_ residues, undercoordinated Pb^0^ sites, and deep‐level traps while suppressing phase segregation; they also optimized the size and coverage of gold nanoparticles deposited on the atomic layer‐deposited (ALD) SnO_2_ layer, thereby enhancing ohmic contact between the top and middle perovskite junctions, minimizing parasitic optical absorption, and suppressing band bending to reduce the carrier recombination barrier at the interface [103]. Thus, using NiO_x_ with the MeO‐2PACz as an HTL to optimize the interface level, the perovskite‐perovskite‐silicon triple‐junction solar cell (MgF_2_/Ag/ITO/SnO_2_/C_60_/PDCl/wide‐bandgap perovskite (1.91 eV)/MeO‐2PACz/NiO_x_/Au nanoparticles (on ALD SnO_2_)/middle‐bandgap perovskite (1.55 eV)/MeO‐2PACz/ITO/<n>a‐Si:H/a‐Si:H/c‐Si(n)/a‐Si:H/<p>a‐Si:H/ITO/Ag) achieved an ultrahigh PCE of 27.06% (1 cm^2^) and a steady‐state PCE of 23.3% (up to 16 cm^2^) (Figure 8e).

So far, research has shown that interface behaviors play critical roles in the performance of perovskite‐based tandem solar cells. In devices with NiO_x_ as the HTL, in addition to improving the quality of perovskite films, a large number of studies have also focused on defect passivation and the suppression of non‐radiative recombination at the interface. Luo et al. innovatively optimized the HTL Me‐4PACz with albendazole (ALB) in inverted wide‐bandgap perovskite solar cells [104]. ALB not only mitigates Me‐4PACz aggregation and promotes the desorption and rearrangement of weakly bound Me‐4PACz molecules to solve the problems of poor wettability, but also uses the nitrogen atom in its benzimidazole ring to coordinate with undercoordinated Pb^2+^ and surface PbI_2_ residues, passivating deep‐level traps (including uncoordinated Pb^2+^‐induced deep trap states and PbI_2_‐derived interfacial defect states) at the buried interface. ALB induced the molecule rearrangement and formed π–π bridges with Me‐4PACz, enabling the four‐terminal perovskite/CIGS tandem cells (using NiO_x_ modified with Me‐4PACz as the HTL) to achieve a high efficiency of 29.06% (Figure 8f). To address the challenges of uncontrollable crystallization and poor buried interface in narrow‐bandgap Pb‐Sn perovskite cells, Yang et al. developed a 2D perovskite seed‐assisted growth strategy [105]. The pre‐deposited 2D F‐PEA_2_PbI_3_SCN seed layer could help inhibit Sn^2+^ oxidation and eliminate interface impurities. The four‐terminal all‐perovskite tandem cell combining the optimized narrow‐bandgap single‐junction device as a bottom cell with the wide‐bandgap perovskite top cell, in which NiO_x_ modified with [4‐(3,6dimethyl‐9H‐carbazol‐9‐yl)butyl]phosphonic acid (Me‐Pacz) is adopted as the HTL. The Me‐Pacz SAM tailors the interfacial energy alignment and passivates surface defects, including oxygen vacancies and undercoordinated Ni ions on the NiO_x_ surface, as well as undercoordinated Pb^2+^ and halide vacancies at the perovskite interface. Ultimately, the device achieved a high efficiency as high as 27.68% (Figure 8g). Fu et al. used piracetam as a crystal modifier, regulating nucleation, promoting preferential growth of [110] crystal planes, and forming one‐dimensional (Pi)PbI_3_ nanostructures at the grain boundaries [106]. The corresponding small‐area (0.07 cm^2^) and large‐area (1.02 cm^2^) all‐perovskite tandem cells with NiO_x_ HTLs modified with Me‐4PACz reached the high PCE values of 28.71% (certified 28.13%) and 28.20%, respectively (Figure 8h).

**FIGURE 9:**
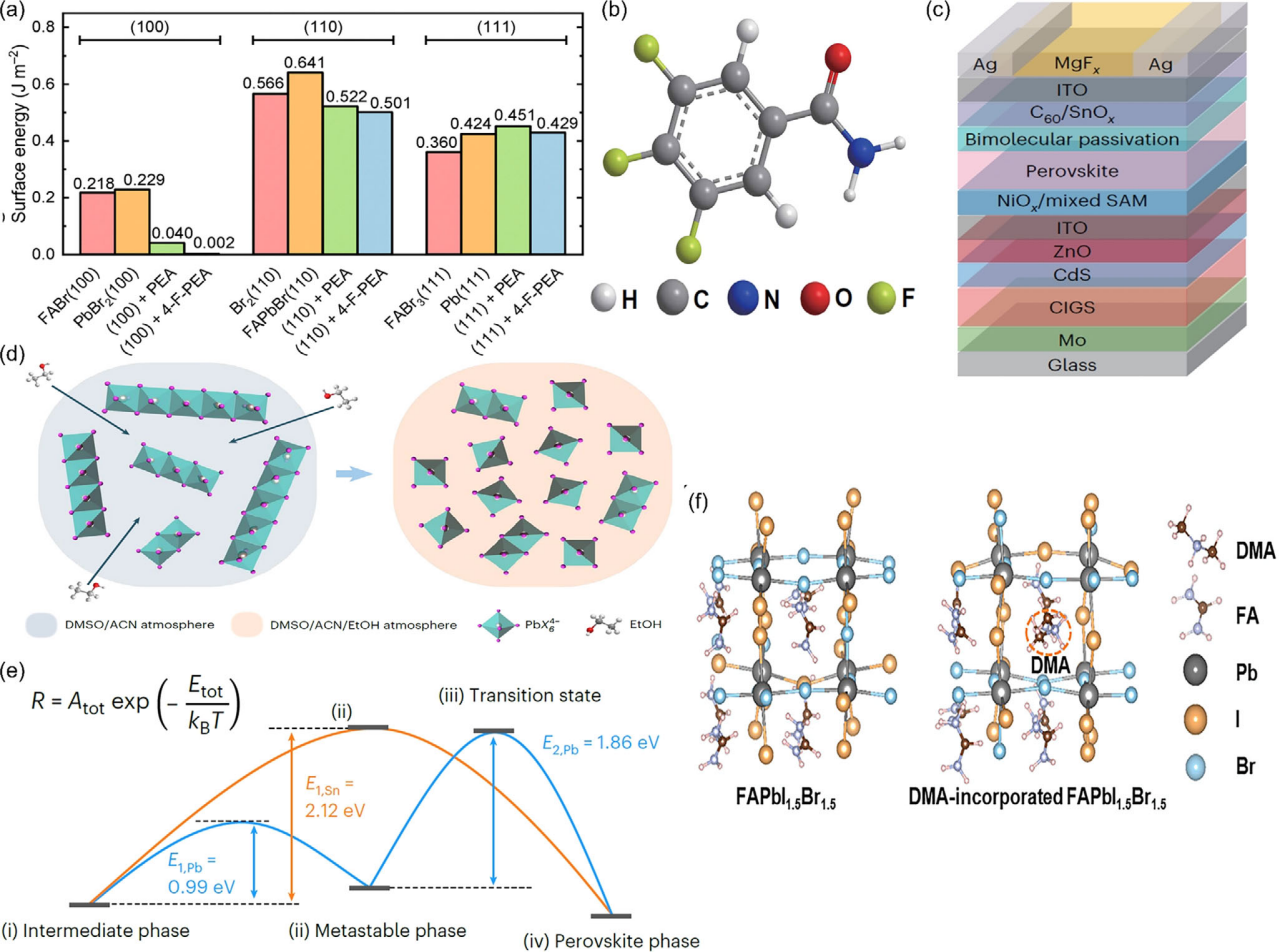
(a) Surface energies of [100, 110], and [111] surfaces considering without and with the treatment of PEA or 4‐F‐PEA. Reproduced with permission [107]. Copyright 2025, Royal Society of Chemistry. (b) Molecular structures of TFBZ. Reproduced with permission [108]. Copyright 2025, The American Association for the Advancement of Science. (c) Schematic of the structure of perovskite/CIGS TSCs. Reproduced with permission [109]. Copyright 2025, Springer Nature Limited. (d) Schematic illustration of the colloidal components after adding EtOH into the perovskite precursor solution. Reproduced with permission [110]. Copyright 2024, Springer Nature Limited. (e) The desorption (*E*_1_) and transition (*E*_2_) barriers from the intermediate to perovskite phase. Reproduced with permission [111]. Copyright 2025, Springer Nature Limited. (f) DFT models of FAPbI_1.5_Br_1.5_ and DMA–Incorporated FAPbI_1.5_Br_1.5_ perovskites. Reproduced with permission [113]. Copyright 2025, Wiley‐VCH.

Recently, Wang et al. utilized NiO_x_ as the foundational inorganic HTL to provide a stable substrate, while modifying its surface with (2‐(4‐(bis(4‐methoxyphenyl)amino)phenyl)‐1‐cyanovinyl)phosphonic acid (MPA‐CPA) as a SAM, which optimizes interfacial energy level alignment and passivates interfacial defects (e.g., undercoordinated Ni^3+^ ions, oxygen vacancies, and surface undercoordinated Pb^2+^ residues), synergizing with NiO_x_ to enhance hole extraction efficiency (Figure 9a) [107]. Meanwhile, they introduced 4‐fluoro‐phenethylammonium (4‐F‐PEA) additive as a template to promote [100]‐oriented wide‐bandgap perovskite growth, reducing deep‐level trap densities (e.g., V_FA_^−^ or Br_Pb_^3−^) and enhancing the binding strength of passivation ligands, ultimately increasing the efficiency of all‐perovskite tandem cells to 28.6%. Jia et al. regulated the crystallization of wide‐bandgap perovskites (without methylammonium) using multifunctional additive 3,4,5‐trifluorobenzoic amide (TFBZ) (Figure 9b), which effectively passivated iodine vacancies and undercoordinated Pb^2+^ defects [108]. This significantly enhanced the perovskite device performance; in particular, the all‐perovskite tandem cells using NiO_x_ HTL modified with Me‐4PACz achieved a high PCE of 29.01% (certified 28.52%). Pei et al. designed a novel passivator, 4‐(2‐aminoethyl)benzenesulfonyl fluoride (TAR‐3), which inhibits passivator desorption under photothermal stress via strong multisite binding (electrostatic interaction and O‐Pb coordination) and enables targeted passivation of multiple defects in wide‐bandgap perovskites (including iodine vacancies (V_I_), undercoordinated Pb^2+^ sites, and halide interstitial defects), while suppressing phase segregation [109]. Meanwhile, they adopted a composite HTL consisting of NiO_x_ as the foundational inorganic layer and a mixed SAM of Me‐4PACz and MeO‐2PACz as the surface modifier (used for optimizing energy level matching). The resulting perovskite/CIGS tandem cell exhibited the certified PCE of 27.35% without degradation after 420 h of continuous operation (Figure 9c).

The use of NiO_x_ HTLs in perovskite‐based tandem solar cells has also supported progress in large‐area fabrication. In 2024, Duan et al. developed a green solvent system consisting of DMSO, acetonitrile (ACN), and ethanol (EtOH), which promotes the formation of DMSO‐PbI_2_ adduct formation (Figure 9d), slows crystallization, and enables uniform large‐area film deposition [110]. By optimizing ion coordination and colloidal behavior, the large‐scale all‐perovskite tandem module (20.25 cm^2^), using NiO_x_ as the HTL and Me‐4PACz to optimize interfacial energy level alignment, exhibited a high efficiency of 23.8%. Recently, Yang et al. reported that Sn‐based and Pb‐based perovskites follow distinct crystallization pathways with different energy barriers (Figure 9e) [111]. By adjusting the DMSO/[Pb+Sn] ratio from 1.85:1 to 2.05:1, the authors balanced the crystallization dynamics of Sn‐Pb perovskites, enabling synchronous crystallization and high‐quality films. This approach yielded the certified all‐perovskite tandem cell efficiency of 28.87% using NiO_x_ with(4‐(7H‐dibenzo[c,g]carbazol‐7‐yl) butyl)phosphonic acid (4PADCB) as the HTL. The SAM molecule 4PADCB is capable of effectively modulating the surface energy levels of NiO_x_ and providing excellent hole transport channels.

In addition to the above work, perovskite/organic tandem solar cells have also attracted considerable attention due to their advantages, such as simple solution preparation, complementary stability, and excellent flexibility. However, halide phase separation induced by light and high bias voltage remains a major obstacle to device performance and long‐term stability [112]. In 2025, Dong et al. proposed a lattice‐enhancement strategy by introducing dimethylammonium ions (DMA^+^) into the A‐site of the perovskite lattice [113]. This modification effectively regulated the tilt of the [PbX_6_]^4−^ octahedron, shortened the Pb‐I bond length, and thereby strengthened lattice stability (Figure 9f). As a result, halide migration and phase separation were significantly suppressed, enabling the perovskite sub‐cells to retain excellent phase stability under intense illumination and high bias. Based on this approach, the fabricated perovskite/organic tandem cells (NiO_x_ with the 2PACz as the HTL, 2PACz was used for optimizing energy level matching) exhibited an efficiency as high as 26.15% (certified 25.34%), and an impressive operational stability of about 1350 h under maximum power point tracking (MPPT).

On the basis of the above, extensive research efforts have targeted these core bottlenecks and achieved breakthroughs. Currently, research on NiO_x_ NP inks has now formed a systematic framework encompassing synthetic process design, doping strategy optimization, performance regulation mechanisms, surface and interface engineering, and expansion of application scenarios. The synergistic effect of metal and non‐metal doping has broken through carrier transport bottlenecks; low‐temperature and green preparation processes have offered solutions to the challenges in large‐scale fabrication; innovations in composite systems have further expanded the functional boundaries of the materials; and the application of the optimized NiO_x_ HTLs in high‐efficiency multi‐junction solar cells, such as perovskite/silicon tandem, all‐perovskite tandem, and perovskite/organic tandem, demonstrates their versatility. These research advances have jointly driven the advancement of this material system from laboratory research to industrial applications. In the future, it will be necessary to further clarify the structure–property–performance relationships among doping, processes, properties, and device function and performance, while paying attention to issues of uniformity control in large‐area fabrications of PSCs.

### 4.2 Sol–Gel Method

#### 4.2.1 Experimental Process and Mechanism

The sol–gel method is a widely employed wet‐chemical approach for preparing metal oxide thin films via liquid‐phase chemical reactions. Its essence lies in the hydrolysis and polycondensation reactions of metal precursors in a solvent to form a sol, which subsequently undergoes gelation to develop a three‐dimensional network structure. Finally, metal oxide thin films can be obtained through the following annealing treatments [114].

**FIGURE 10:**
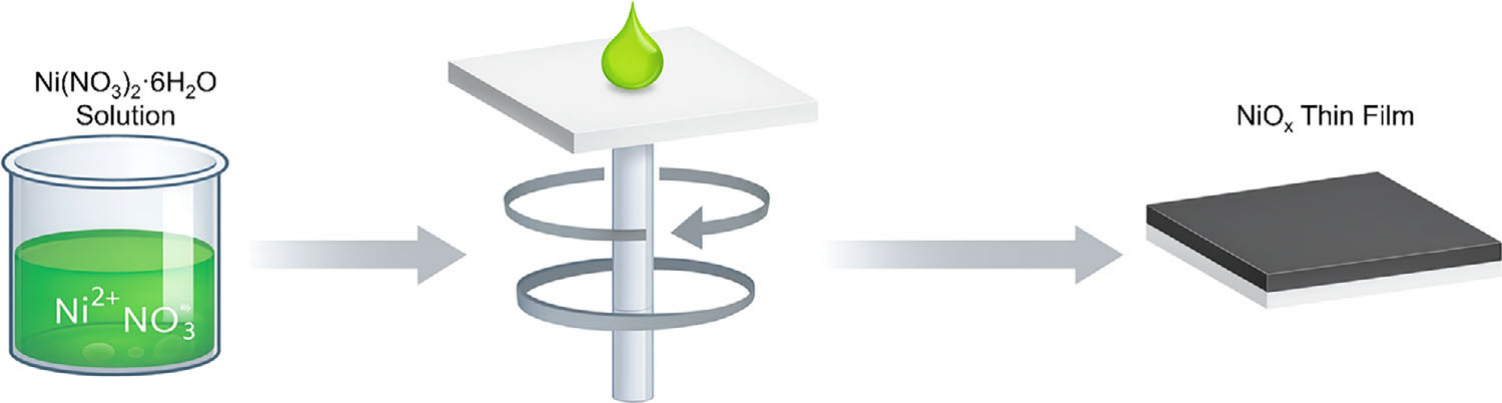
Schematic diagram of a typical sol–gel method. Ni(NO_3_)_2_·6H_2_O solution is mixed with the stabilizers to form a homogeneous sol; thereafter, the sol is spin‐coated and is then annealed to form NiO_x_ film.

As for the NiO_x_ HTLs prepared using the sol–gel method, their process could begin with the preparation of NiO_x_ precursors, where the nickel‐based salts (such as Ni(NO_3_)_2_·6H_2_O or Ni(CH_3_OCO)_2_·4H_2_O) are mixed with the stabilizers (e.g., monoethanolamine) in an organic solvent (e.g., ethanol, ethylene glycol, and isopropyl alcohol) to form a homogeneous sol (Figure 10) [115, 116, 117, 118, 119]. The obtained sol is then coated onto the substrate via spin‐coating or spray‐coating method, followed by annealing treatment at above 300°C to remove organic components and promote crystallization [114, 119, 120, 121]. Thereafter, the high‐quality NiO_x_ thin films could be obtained. Clearly, this method features the significant advantage of facile synthesis process for obtaining high‐quality NiO_x_ thin films.

#### 4.2.2 Existing Issues

It should be noted that the sol–gel method still faces several scientific issues and technical bottlenecks when preparing high‐quality NiO_x_ thin films as the HTLs to meet the requirements of highly efficient and stable PSCs.

The sol‐gel process normally requires high‐temperature annealing treatment (even though the temperature is not that high), which could still limit its applicability [22, 122]. In particular, this high‐temperature condition is incompatible with flexible substrates and roll‐to‐roll mass production technology, and it is also unsuitable for ITO‐coated substrates, which are widely employed in the research of PSC devices. In addition, although high‐temperature annealing treatment could improve crystallinity of the obtained thin films, it could also introduce a large number of surface defects within the obtained thin films. The difficulty in controlling film uniformity and defects would lead to the enhanced charge carrier recombination between NiO_x_ thin films and perovskite active layers, reducing the *J*_sc_ and *FF* values of the obtained PSCs. Previous reports have shown that the pinholes and local defects in sol–gel‐derived NiO_x_ HTLs formed due to uneven precursor distribution or differential annealing rates could not only cause low *J*_sc_ and *FF* values in the related PSC devices by exacerbating interfacial charge accumulation and non‐radiative recombination but also lead to significantly inferior long‐term stability compared to their doped or surface‐modified counterparts [21, 22, 114, 123, 124, 125]. These issues could collectively restrict the wide application of the sol‐gel method for NiO_x_ thin films in high‐performance and stable NiO_x_‐based PSCs; in this regard, various strategies have been developed for addressing these issues for moving this field forward.

#### 4.2.3 Progress in PSCs With Sol–Gel‐Prepared NiO_x_ HTLs

##### 4.2.3.1 Doping Engineering: Modulating Conductivity, Crystallization, and Energy Levels

**FIGURE 11:**
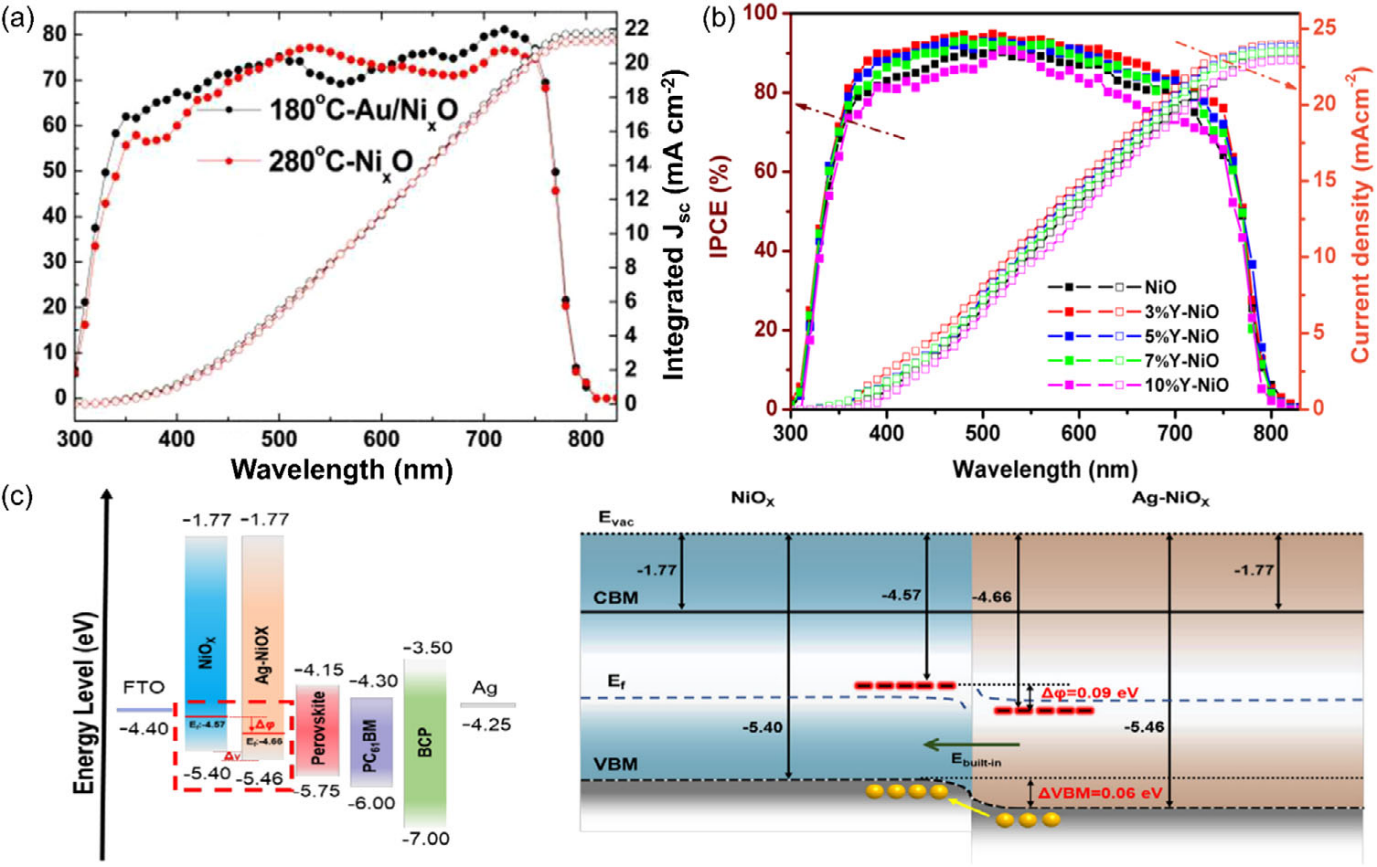
(a) IPCE spectra and integrated *J*_sc_ curves of devices with optimally sintered Au nanoparticles‐embedded NiO_x_ HTL and NiO_x_ HTL. Reproduced with permission [126]. Copyright 2018, Royal Society of Chemistry. (b) IPCE spectra of x% Y‐NiO (x = 0, 3, 5, 7, and 10) based perovskite solar cells. Reproduced with permission [127]. Copyright 2018, Elsevier. (c) Corresponding energy‐level diagram of hybrid NiO_x_ ‐based inverted PSC and energy‐level structures of NiO_x_ and Ag‐doped NiO_x_ thin films. Reproduced with permission [128]. Copyright 2023, Wiley‐VCH.

The core mechanism of the doping strategy lies in fundamentally altering the band structure, carrier concentration, and crystallization kinetics of NiO_x_ by introducing foreign ions. In 2014, Zhu et al. synthesized high‐quality NiO_x_ thin films via the sol‐gel method and applied them as HTLs in high‐performance PSCs [114]. As for this sol‐gel method, doping is a key strategy to improve NiO_x_ thin‐film quality and its physical properties. By introducing different doping elements and innovating doping mechanisms, researchers have effectively overcome the limitations of NiO_x_ thin films prepared by this method in terms of their electrical conductivity, energy level alignment, and high‐temperature fabrication process. In 2018, Hou et al. proposed the catalytic metal‐induced crystallization (c‐MIC) mechanism; in detail, they incorporated Au dopants into NiO_x_ precursors and employed the sol‐gel method to realize the low‐temperature sintering, reducing the sintering temperature of NiO_x_ thin films from 280°C to 180°C (Figure 11a) [126]. Due to the method optimization, both the flexible and rigid PSCs could be obtained; the related devices showed the high efficiencies of 15.9% and 19.0%, respectively, and exhibited high stability exceeding 1200 h. This induced‐crystallization approach could effectively reduce the residual organic ligands and retain the surface NiOOH groups, thereby enhancing the hole collection capability of the obtained NiO_x_ HTLs. However, the incorporation of Au nanoparticles could increase the material costs, limiting its large‐scale application. In the same year, Hu et al. prepared Y‐doped NiO_x_ thin films via the sol‐gel method and applied them in the inverted PSCs [127]. Y^3+^ substitution for Ni^2+^ expanded the lattice and increased the *V*_O_ concentration, thereby enhancing the *p*‐type conductivity of the obtained thin films. Meanwhile, this doping process also improved the interfacial contact between NiO_x_ HTLs and the perovskite active layers and reduced charge carrier recombination at the interfaces. Finally, as shown in Figure 11b, the relevant devices using NiO_x_ HTLs with 5% Y doping exhibited the best incident‐photon‐to‐electron‐conversion efficiency (IPCE) and integrated current, achieving a high PCE of 16.31%, which corresponds to a 27.62% improvement ratio as compared to the devices with undoped NiO_x_ HTLs. This study provides a reference for the application of rare earth metal doping in the sol–gel‐prepared NiO_x_ systems for improving device performance of the related PSCs.

In 2023, Wang et al. reported a strategy of forming p/p^+^ homojunctions via applying Ag doping in NiO_x_ thin films, which was employed as the HTLs in the inverted PSCs [128]. Ag^+^ doping could reduce the VBM of NiO_x_ to 5.46 eV (Figure 11c), achieving optimized energy level alignment with perovskite active layers. Meanwhile, the built‐in electric field of the *p*/*p* ^+^ homojunction could also promote charge separation and suppress charge carrier recombination. The PSC devices based on the Ag‐doped NiO_x_/NiO_x_ (*p* ^+^/*p*) homojunction HTLs achieved a high PCE of 19.25%, which represents a significant improvement compared to the devices based on pure NiO_x_ HTLs (with a PCE of 18.83%), and retained 90% of their initial efficiency after even 30 days. This work expanded the dimensions of doping strategies in the sol‐gel method through the homojunction design; however, the issue of Ag^+^ migration may affect the long‐term stability of PSC devices, which requires further research to address this key issue. Therefore, it should be noted that even though the doping process is an effective strategy for improving the quality and physical properties of NiO_x_ thin films and thus the related PSC device performance, there are still some issues that need further investigation.

##### 4.2.3.2 Interface Modification: Passivating Defects and Aligning Energy Levels

**FIGURE 12:**
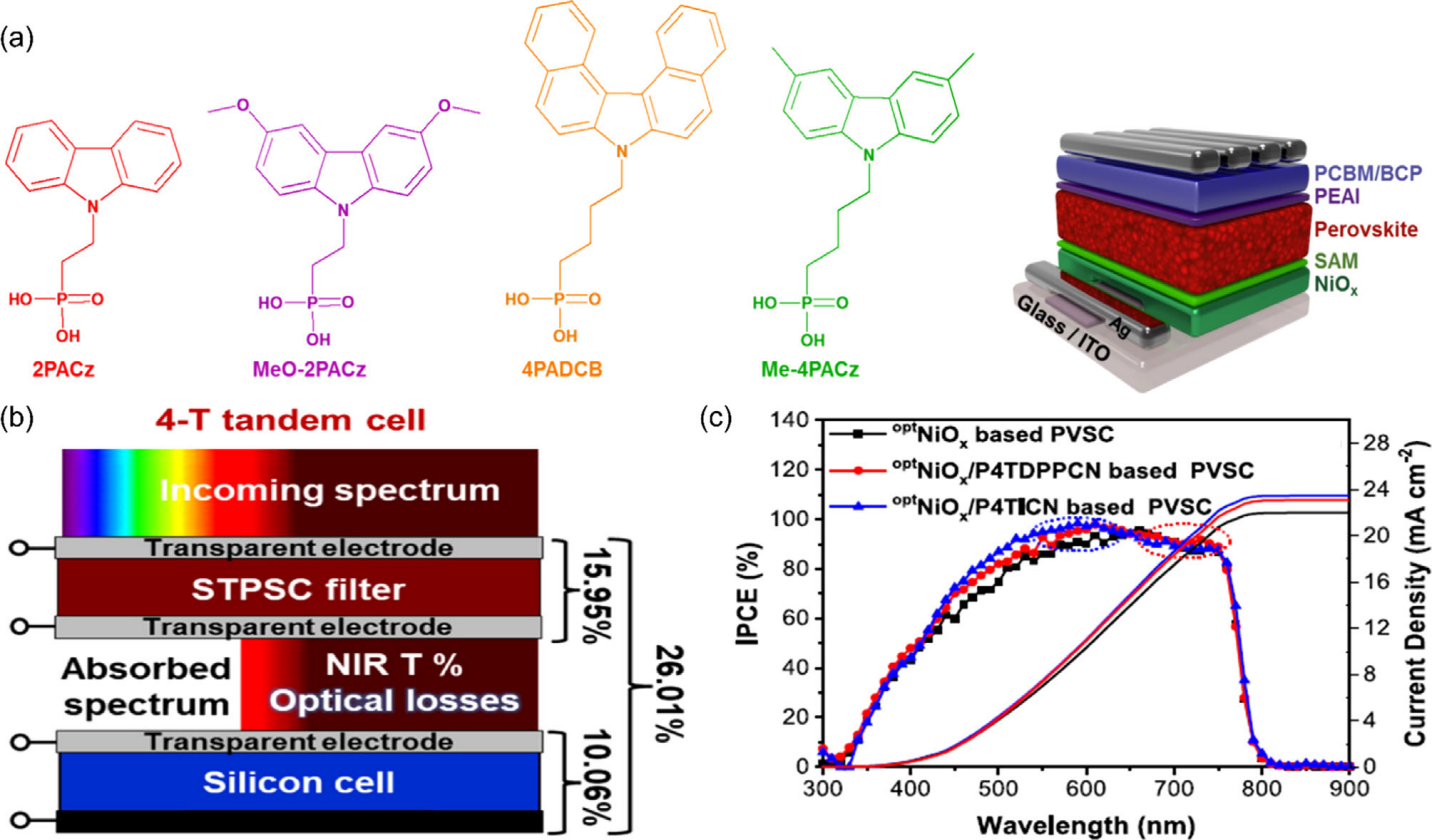
(a) Chemical structures of SAMs and device architecture used in this study. Reproduced with permission [129]. Copyright 2024, Elsevier. (b) A schematic illustration showing the testing scheme for the 4T ST‐PSC/SiSC stacked tandem solar cells. Reproduced with permission [130]. Copyright 2023, Elsevier. (c) IPCE spectra of FTO/opt NiO_x_ with or without polymer modification/MAPbI_3_/PC61BM/BCP/Ag PVSCs. Reproduced with permission [131]. Copyright 2023, American Chemical Society.

Beyond doping strategy, interface modification serves as another crucial approach to enhance the quality and physical properties of NiO_x_ thin films prepared by the sol‐gel method. By passivating defects and optimizing energy level alignment, this strategy effectively compensates for the limitations of pure doping in interface regulation of NiO_x_ thin films. For instance, Shi et al. modified the sol–gel‐derived NiO_x_ thin films using SAMs (MeO‐2PACz and 4PADCB), aiming to enhance the indoor light‐harvesting efficiency of the related PSCs [129]. Through Lewis acid–base interactions, SAMs could passivate Pb^2+^ defects at the NiO_x_/perovskite interface and reduce the trap state density [132]. Meanwhile, the introduced SAMs could also regulate the energy levels of NiO_x_ thin films and enhance hole extraction capability. NiO_x_‐based PSC devices modified with various SAMs, as shown in Figure 12a, exhibited a high indoor IPCE of up to 42.05% under a 3000‐K LED light source. Tyagi et al. adopted a composite structure strategy by first spin‐coating a sol‐gel‐derived NiO_x_ dense layer and then spray‐depositing NiO_x_ colloidal particles to form a porous layer [130]. The authors showed that the dense NiO_x_ layer effectively suppresses electron transport, while the porous‐structured NiO_x_ layers increase the contact area with the perovskite active layers, facilitating charge extraction in the devices. Meanwhile, high crystallinity of NiO_x_ thin films reduces light absorption loss and improves transmittance in the near‐infrared region, also beneficial for the related PSC device performance. Single‐junction PSCs based on this structure achieved a PCE of 15.9%, while the tandem cells composed of these and silicon cells exhibited a high efficiency of 26.0% (Figure 12b). This study opens a path for the application of sol‐gel‐derived NiO_x_ in tandem PSC devices, though the preparation consistency of the bilayer structure still needs further improvement.

##### 4.2.3.3 Precursor Optimization: Governing Film Crystallization and Phase Composition

In the sol–gel method for NiO_x_ thin films, precursor ratio normally serves as a core parameter to regulate reaction progression and film quality, directly influencing the phase composition, defect density, and optoelectronic properties of NiO_x_ HTLs. In 2024, Kuo et al. systematically investigated the impact of the molar ratio of nickel acetylacetonate (Ni(acac)_2_) to diethanolamine (DEA) (ranging from 1:0.6 to 1:1.4) on the quality and physical properties of NiO_x_ thin films [131]. They introduced cyano‐containing photovoltaic polymers (such as pBαCN, P4TDPPCN, and P4TICN) as interlayers between NiO_x_ and MAPbI_3_ perovskite active layer to construct high‐performance inverted PSCs (Figure 12c). Studies revealed that a lower DEA ratio (1:0.8) promoted the formation of the highly conductive Ni_2_O_3_ phase in NiO_x_ films, reduced NiOOH defects, and thereby enhanced film conductivity. Further ultraviolet (UV) treatment increased Ni^3+^ content, optimized energy level alignment, and minimized interfacial energy loss. Cyano‐containing polymers like P4TICN improved interface wettability and contact uniformity through chemical interactions between their carbonyl groups (C═O) and Pb^2+^ in the perovskite active layers, effectively reducing trap state density (*n*_trap_) and charge recombination resistance (*R*_rec_). Meanwhile, the polymers' inherent light absorption contributed additional photocurrent, thus boosting the *J*_sc_ values of the obtained PSCs. Ultimately, the optimized device achieved a high PCE of 21.43%, with a *J*_sc_ exceeding 24 mA/cm^2^ and an *FF* approaching 80%. This study systematically established the relationship between precursor ratio, film structure, and device performance; however, the combined effects of different nickel sources and amine‐based stabilizers still require further investigation.

To date, NiO_x_ thin films prepared by the sol–gel method have achieved significant progress in low‐temperature sintering, conductivity enhancement, energy level alignment, and interfacial defect passivation through strategies such as element doping, interface engineering, structural design, and precursor optimization. These advancements have driven the efficiency of PSCs to significantly increase from the baseline level in the devices based on the pure NiO_x_ HTLs to over 21%, while simultaneously balancing excellent stability and the feasibility of large‐area and/or flexible device fabrications. Future research could further integrate multi‐dimensional modification strategies to optimize film crystallinity and interfacial compatibility of NiO_x_ HTLs and expand their applications in fields such as flexible devices and large‐area tandem solar cells.

### 4.3 Solution Combustion Method

#### 4.3.1 Experimental Process and Mechanism

**FIGURE 13:**
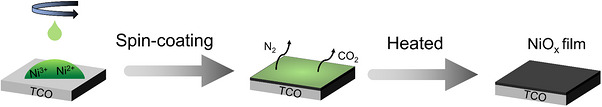
A typical chemical combustion method involves mixing acetylacetone as fuel with nickel nitrate to form a precursor, which is then spin‐coated onto TCO and heated to react and form NiO_x_ thin film.

The solution combustion method is a low‐temperature synthesis technique based on solution‐phase redox reactions. Its essence lies in the rapid synthesis of target materials and thin‐film fabrication via self‐propagating exothermic reactions between fuel and oxidizer in solution (Figure 13) [133]. As shown in Equation 3, its reaction principle is that fuel (e.g., acetylacetone) and metal nitrate (e.g., nickel nitrate) undergo a vigorous redox reaction at low temperature, releasing a large amount of heat to promote the crystal growth [134, 135, 136, 137].

(3) $$\begin{array}{cc} & \mathrm{Ni}{\left(NO_{3}\right)}_{2}\cdot 6H_{2}O\;+\;CH_{3}\mathrm{COC}H_{2}\mathrm{COC}H_{3}\\ & \;\to \;\mathrm{Ni}O_{X}\left(s\right)+H_{2}O↑+N_{2}↑+\;CO_{2}↑ \end{array}$$

As for this method, the ratio of fuel to oxidizer exerts a significant regulatory effect on reaction intensity and product quality. For instance, deviating from the stoichiometric ratio may affect crystal growth kinetics by altering combustion temperature and gas release [138, 139]. Its process generally involves first dissolving metal salts and fuel in proportion to form a homogeneous solution. The choice of fuel determines the intensity of the exothermic reaction and may also affect the viscosity of the solution as well as the specific pathway of subsequent combustion. Subsequently, methods such as spin‐coating are used to coat the solution drop onto the substrate, forming a uniform precursor thin film. Finally, heating is applied to initiate the combustion reaction. The reaction is self‐heating, burning off organics while crystallizing inorganics into a thin film, eliminating the high‐temperature annealing step of the traditional methods. Gases generated during combustion (such as N_2_ and CO_2_) would leave pores in the film, forming a porous structure. This issue can be precisely controlled by changing the type of fuel or adjusting the fuel amount [136, 137, 140].

#### 4.3.2 Existing Issues

In meeting the demand for high‐quality NiO_x_ thin film as efficient HTLs, this solution combustion method still faces multiple scientific issues and technical bottlenecks. Notably, the doping process is also a widely applied strategy to improve the physical properties of the obtained NiO_x_ thin films using this method. However, the uniformity of doped ions is difficult to precisely regulate, as the self‐propagating nature of the combustion reaction could lead to large local temperature gradients, which easily cause agglomeration of doped ions (such as Cu^2+^ and Li^+^). For instance, in the case of Cu‐doped NiO_x_ thin films, excessively high concentrations can form the Cu clusters in the films, which could scatter incident light, resulting in reduced transmittance and impaired charge carrier mobility [141, 142, 143, 144]. Notably, high‐temperature requirements (for example, more than 400°C for sol–gel method and 300°C for the solution combustion method) and reaction‐induced defects (combustion‐generated pinholes) remain common barriers to substrate compatibility across methods [145, 146, 147]. These issues directly limit the use of NiO_x_ HTL synthesized by the solution combustion method, which would require some breakthroughs through methods such as optimizing the fuel/oxidizer ratio and introducing interface modification [140, 148].

#### 4.3.3 Progress of PSC Performance Optimization

As an important solution‐based chemical technique for preparing NiO_x_ HTLs, the solution combustion method has gained extensive attention in the field of PSCs due to its unique reaction mechanism and process advantages. Its core principle is that the oxidizer and fuel in the precursor solution undergo a self‐exothermic reaction after ignition, rapidly forming a metal‐oxide‐metal (M‐O‐M) lattice, with the reaction temperature usually being controlled at 200–300°C [67]. Compared with the sol‐gel process, the solution combustion method does not require a lengthy gelation process, featuring a faster reaction rate and lower energy consumption, thus providing a new approach for the efficient preparation of high‐crystallinity NiO_x_ thin films.

##### 4.3.3.1 Doping Engineering: Modulating Conductivity, Crystallization, and Energy Levels

**FIGURE 14:**
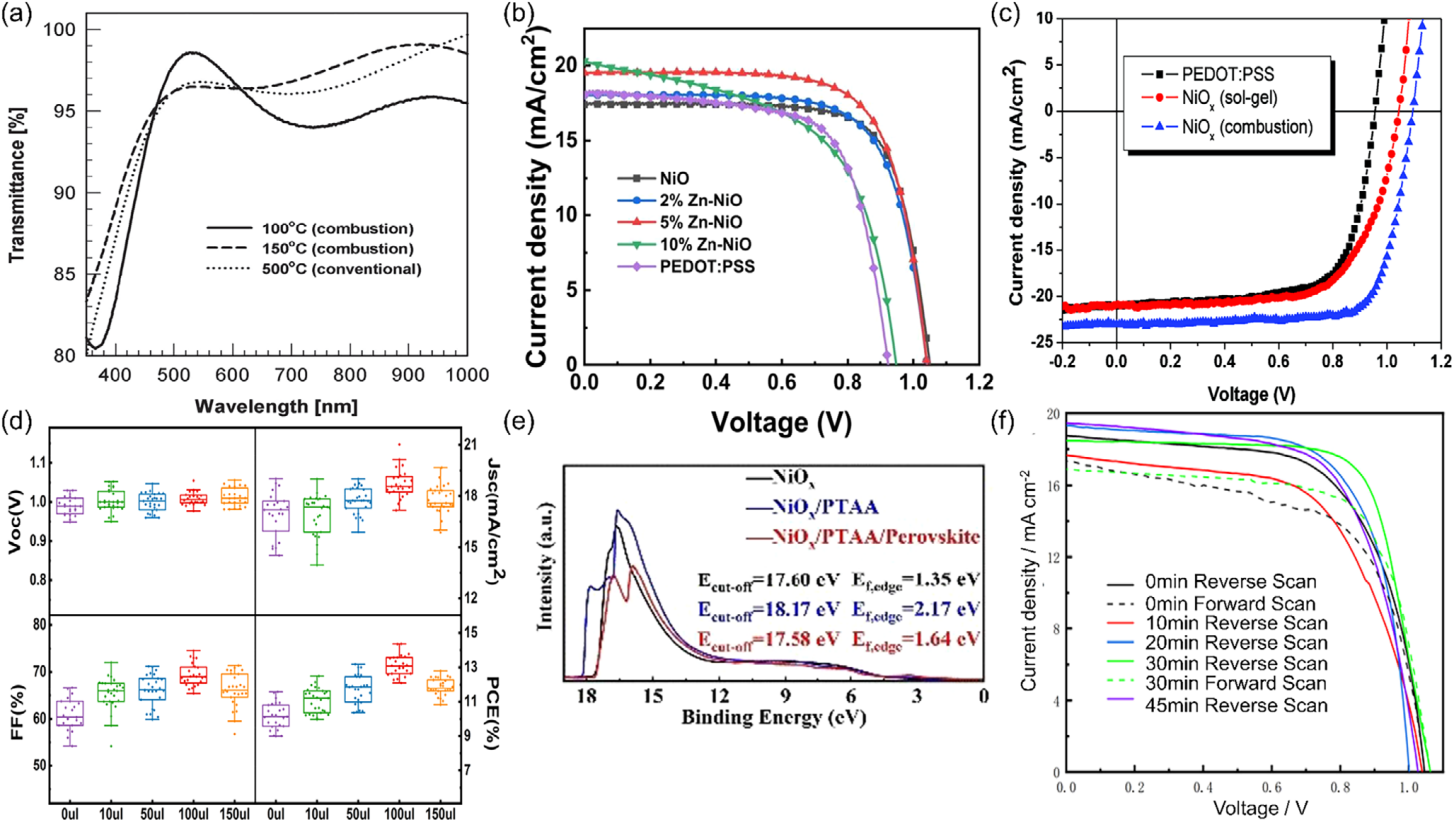
(a) Transmittance spectra of the Cu:NiO films prepared through combustion and conventional methods. Reproduced with permission [133]. Copyright 2015, Wiley‐VCH. (b) *J‐V* characteristics of PSCs made with NiO_x_, Zn:NiO_x_, and PEDOT:PSS (control) HTLs. Reproduced with permission [136]. Copyright 2021, MDPI. (c) *J‐V* curves of perovskite solar cells based on different HTLs. Reproduced with permission [148]. Copyright 2018, Wiley‐VCH. (d) Photovoltaic device parameters of the inverted devices based on different acetylacetone contents of the NiO_x_ HTLs. Reproduced with permission [149]. Copyright 2020, Springer Science Business Media. (e) UPS spectra of NiO_x_, NiO_x_/PTAA, and NiO_x_/PTAA/LDRP perovskite films. Reproduced with permission [150]. Copyright 2023, Wiley‐VCH. (f) *J‐V* curves of PSCs based on different bilayer NiO_x_ HTLs under varied deposition time. Reproduced with permission [151]. Copyright 2022, Springer Science Business Media.

In 2015, Jung et al. first reported the preparation of Cu‐doped NiO_x_ (Cu:NiO_x_) thin films via the solution combustion method at 150°C [133]. The authors utilized the self‐exothermic reaction between Ni(NO_3_)_2_ and CH_3_COCH_2_COCH_3_ to form high‐crystallinity NiO_x_ thin films without high‐temperature sintering; the obtained films show a high electrical conductivity of 1.25 × 10^−3^ Scm^−1^, which is about 1.6 times higher than that of the samples prepared by the traditional sol‐gel method (annealed at 500°C). Additionally, the obtained NiO_x_ thin films exhibited higher transparency behavior in the 600–950 nm wavelength range (Figure 14a), which is beneficial for enhancing light absorption efficiency of perovskite active layers. The PSCs based on the HTLs through the method exhibited excellent device performance (the maximum PCE could reach 17.74%), while the champion PCE of the flexible PSCs reached up to 13.4%, laying a foundation for the low‐temperature preparation of NiO_x_ HTLs and facile fabrication of high‐performance PSCs. This study confirmed the advantages of the solution combustion method in the low‐temperature and efficient preparation of high‐efficiency PSCs. However, the control of Cu‐doping uniformity still needs further improvement, while the issue of performance degradation in large‐area devices also remains unsolved. With the deepening of research, doping modification has become a key strategy to optimize the quality and properties of combustion‐synthesized NiO_x_. In 2021, Thiruchelvan et al. further modified the solution‐combustion‐synthesized NiO_x_ thin films via applying 5% Zn doping (Figure 14b), which increased the Ni^3+^/Ni^2+^ ratio, enhanced electrical conductivity, and improved charge extraction efficiency (photoluminescence‐related lifetime shortened from 10.1 ns to 9.3 ns) [136]. The relevant PSC devices based on MAPbI_3_ perovskite layers achieved a high PCE of 14.87% and exhibited improved stability under 40% relative humidity, verifying the effectiveness of the doping strategy in the solution combustion method. This work successfully established Zn as a viable dopant to enhance the performance of combustion‐processed NiO_x_ HTLs, expanding the library of effective elements beyond Cu. The observed improvement in electrical conductivity and device efficiency underscores the promise of this approach. However, similar to the previously reported doping process, such as Cu doping, the incorporation of foreign ions could introduce interfacial contact and energy level alignment challenges at the NiO_x_/perovskite junction. Therefore, future optimization of Zn‐doped NiO_x_ and development of new doping elements, as well as doped metal oxides in general, should combine bulk property enhancement with tailored interface modification strategies to fully unlock their potential.

##### 4.3.3.2 Process Parameter Optimization: Regulating Crystallization, Microstructure, and Energy Levels

Systematic optimization of process parameters has further unleashed the potential of the solution combustion method. Liu et al. further optimized the combustion process to synthesize pure‐phase NiO_x_ thin films at 250°C, with the better energy level alignment with the perovskite layers than that prepared by sol–gel method (*W*_F_ of NiO_x_ is 5.43 eV, and VB of perovskite is 5.4 eV) [148]. Additionally, the obtained NiO_x_ thin films exhibited a higher Ni^3+^/Ni^2+^ ratio, which significantly improved their electrical conductivity and charge extraction capability. By combining with the modified two‐step method for preparing perovskite layers, specifically MA_1‐y_FA_y_PbI_3‐x_Cl_x_, the PCE of the related PSC devices using NiO_x_ HTLs could exceed 20%, as shown in Figure 14c. Meanwhile, the device stability was also significantly improved, retaining 93.7% of its initial efficiency after being stored in air for 30 days. In terms of the process parameters, there is also the regulation of fuel concentration that has been proven to improve NiO_x_ thin‐film quality and the related device performance. Liu et al. focused on the influence of fuel concentration and found that 100 µL/10 mL was the optimal concentration for optimal NiO_x_ HTLs (Figure 14d) [149]. At this concentration, the obtained NiO_x_ thin film exhibited the highest crystallinity and the lowest surface roughness (just about 9.63 nm), which promoted the formation of larger‐grain perovskite layers (about 230 nm) with reduced trap states. The maximum PCE of the relevant PSC devices reached up to 14.37%. The research further revealed that the fuel concentration could influence the microstructure of NiO_x_ thin films by regulating the exothermic intensity of the combustion reaction. In 2023, Xie et al. optimized the VBM of NiO_x_ thin films by adjusting the fuel/oxidizer ratio in the solution combustion reaction (Figure 14e), reducing the energy level difference with low‐dimensional perovskite layer to 0.1 eV [150]. This could effectively suppress non‐radiative recombination at NiO_x_/low‐dimensional perovskite interfaces, with the *V*_OC_ increasing from 1.05 V to 1.12 V and the PCE reaching 12.25%, highlighting the flexibility in energy level regulation.

##### 4.3.3.3 Interface and Structural Engineering: Optimizing Charge Transport and Energy Level Matching

**FIGURE 15:**
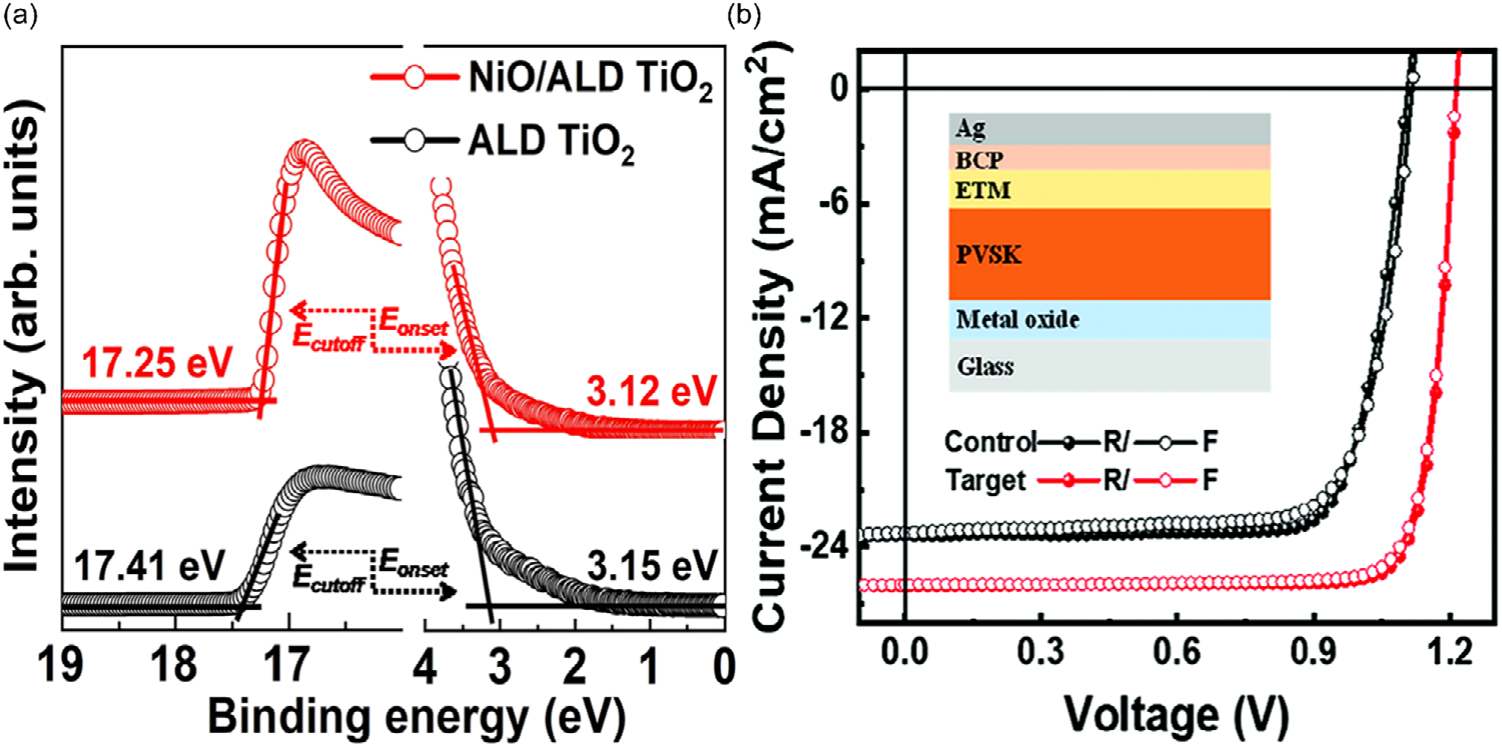
(a) UPS spectra of ALD‐prepared TiO_2_ and NiO/ALD‐TiO_2_ ETLs, along with the estimated VBM onset (*E*_on‐set_) and photoemission cutoff energy boundary (*E*_cut‐off_) values via linear fittings, respectively. Reproduced with permission [152]. Copyright 2020, Elsevier. (b) *J–V* curves of inverted PSCs using NiO_x_ HTLs without (control) and with additives (target). Reproduced with permission [153]. Copyright 2024, Wiley‐VCH.

In 2022, Song et al. proposed the bilayer NiO_x_ HTL structure; that is, the bottom NiO_x_ layer was prepared by the solution combustion method, and the top NiO_x_ layer was prepared by the hydrothermal method [151]. Optimal PSC device performance was achieved with a further 30‐min hydrothermal treatment on the bilayer NiO_x_ HTLs (Figure 14f). The research further demonstrated that the bilayer structure could effectively improve interfacial contact and charge transport pathways in the relevant PSC devices, increasing the *FF* to 0.78 and the PCE to 14.3%. This further unleashes the performance improvement potential of solution combustion‐synthesized NiO_x_ in PSC devices. Furthermore, the bilayer structure can further expand the application scope of NiO_x_ thin films in various other types of photovoltaic devices. Chai et al. combined the NiO_x_ film prepared by the solution combustion method with TiO_2_ film synthesized using atomic layer deposition (ALD) through structural design and interface regulation, thereby constructing a NiO_x_/ALD‐TiO_2_ bilayer. The authors also successfully employed this obtained bilayer as the efficient ETL in the PSC applications [152]. Moreover, the NiO_x_ layer could serve the buffer layer, which significantly reduces the content of hydroxyl (‐OH) species on the surface of ALD‐grown TiO_2_ layer. Meanwhile, the band bending effect generated at the interface between NiO_x_ and TiO_2_ layers optimizes the band structure of the bilayer ETL, which increases from 3.81 eV (for pure ALD‐grown TiO_2_) to 3.97 eV (Figure 15a). This change raises the offset between the bilayer structure and the conduction band minimum (CBM) of CsPbIBr_2_ perovskite active layer from 0.33 eV to 0.46 eV, which can effectively block the reverse backflow of electrons and significantly reduce energy loss. The constructed all‐inorganic CsPbIBr_2_ perovskite solar cell with this NiO_x_/ALD‐TiO_2_ bilayer working as the ETL exhibited excellent device performance, with a high PCE of 9.71%. Currently, more studies have focused on the defect regulation of NiO_x_ HTLs, and some significant breakthroughs have been achieved. Huang et al. employed combustion‐synthesized NiO_x_ as HTL in the inverted PSCs. They further optimized the perovskite active layer through an *in‐situ* passivation technology. This synergistic approach of using a high‐quality NiO_x_ HTL combined with a superior perovskite layer resulted in a significant reduction of the interfacial defect state density and maintained high electrical conductivity (2.1 × 10^−3^ S cm^−1^) [153]. Ultimately, the PCE of the relevant inverted PSCs could exceed up to 26.7% (certified 26.09%; Figure 15b), setting a record for NiO_x_‐based PSCs with NiO_x_ being prepared by the solution combustion method. It is evident that, through multi‐dimensional optimization, the solution combustion method has become the core technology for preparing NiO_x_ HTLs for high‐performance PSCs.

As can be seen from the research work as summarized above, the development of the solution combustion method is built on its core advantage of a low‐temperature process and a high‐quality product. Through strategies such as optimization of process parameters (e.g., annealing temperature, fuel concentration), doping modification (Cu, Zn), and structural design (bilayer), sustained breakthroughs in PCE applications have been achieved, increasing the PCE values from 17.74% in 2015 to 26.7%. Meanwhile, the device stability and substrate compatibility have been significantly improved. Therefore, leveraging these technical routes to further tap into the potential of the solution combustion method could provide critical support for further industrialization of PSCs.

### 4.4 Chemical Bath Deposition

#### 4.4.1 Experimental Process and Mechanism

**FIGURE 16:**
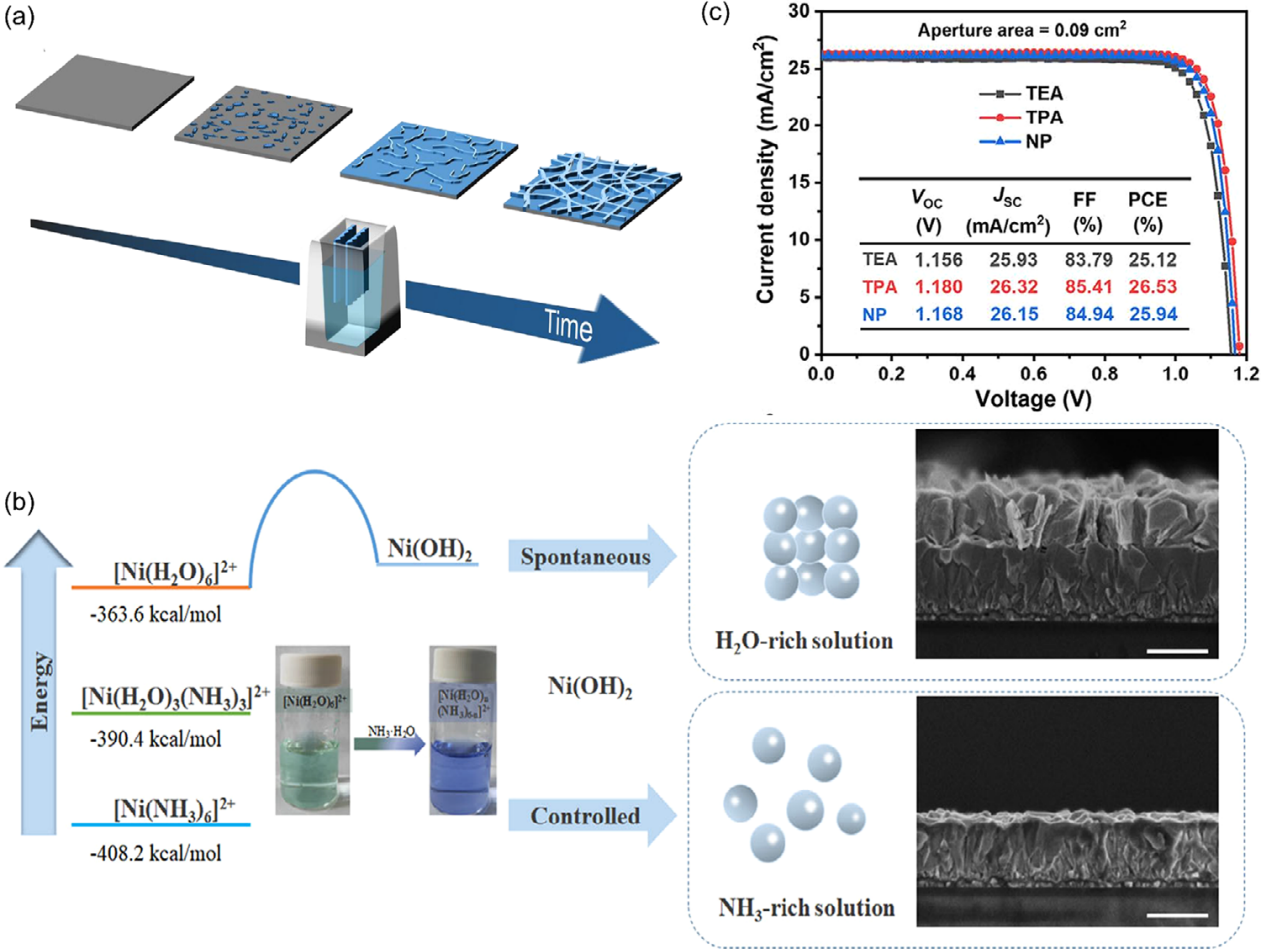
(a) Schematic illustration of the chemical bath deposition (CBD) process for depositing mesoporous Ni hydroxide/oxyhydroxide films on FTO coated glass before dehydration. Reproduced with permission [154]. Copyright 2018, Elsevier. (b) Schematic diagram of the relative energy states of the Ni‐based complexes and their influence on hydrolysis kinetics and quality of the fabricated films. Reproduced with permission [159]. Copyright 2024, Wiley‐VCH. (c) The champion J–V curves for the TEA, TPA, and NP‐based devices. Reproduced with permission [160]. Copyright 2025, Wiley‐VCH.

As shown in Figure 16a, chemical bath deposition (CBD) method is a typical wet chemical method for *in‐situ* growth of thin films on the substrates through chemical reactions in solutions. Its essence lies in utilizing soluble precursors to undergo hydrolysis, complexation, or redox reactions in a liquid‐phase environment, generating insoluble products that deposit onto the substrate surface [67, 154]. The key essence of its chemical reaction lies in the controllable reaction of ions in the solution. For example, amino alcohol ligands are used to regulate the release rate of Ni^2+^, enabling uniform deposition of Ni(OH)_2_ precursors, which are then converted to NiO_x_ after annealing treatment [67]. Related reactions can be promoted and tuned by regulating the pH ratio, temperature level, and other factors, which could also aid in removing impurities and facilitating crystallization [155, 156].

The CBD technique demonstrates significant advantages in the synthesis of inorganic charge transport layers for PSCs, owing to its cost‐effectiveness, simple process, and scalability [157]. This method enables large‐area uniform thin‐film deposition through solution immersion, as evidenced in *n‐i‐p* structured PSC devices, where CBD‐derived SnO_2_ ETLs have been successfully implemented. By precisely adjusting precursor composition, the film stoichiometry can be optimized, leading to enhanced device performance [158]. Compared to conventional solution‐based techniques such as sol‐gel and combustion methods, CBD offers a more promising route for large‐scale production of *p*‐type NiO_x_ HTLs. Although anisotropic growth of intermediate phases remains a challenge in forming planar NiO_x_ via CBD, overcoming this limitation would substantially advance the industrialization of the relevant inverted *p‐i‐n* PSCs [154].

#### 4.4.2 Existing Challenges

It should be noted that, despite some advantages, the CBD method normally suffers from the key issue of severe material waste. In addition, the excessive precipitation could form in the solution during the relevant reaction, and a great amount of materials would deposit on the reactor walls, thus impairing the film uniformity [161]. Additionally, the impurities in the precursor, such as NO_3_^−^ and OH^−^, could easily diffuse into the formed NiO_x_ thin films, forming defects that would increase the non‐radiation recombination and exacerbate the redox reactions between Ni^3+^ and the perovskite active layers, significantly reducing the PSC device performance and stability [78, 162].

Low crystallinity and high dispersibility of the obtained NiO_x_ thin films prepared by this method are also key challenges. Calcination below 270°C normally leaves the residual Ni(OH)_2_, reducing the conductivity of the obtained thin films. However, if the temperature is too high, the related NiO_x_ NPs would agglomerate and fail to disperse properly, which could not form a uniform thin film [163, 164]. Moreover, NiO_x_ itself could exhibit relatively poor electrical conductivity (10^−4^ S cm^−1^) and thus require improvement through some strategies such as doping engineering. However, achieving the uniform distribution of dopant elements such as Li^+^ and Cu^2+^ in NiO_x_ precursor and the related thin films is highly challenging, which easily leads to the formation of local defects in the obtained thin films [165]. Additionally, there is also the issue regarding the interfacial defects, where Ni^3+^ cations on the surface of NiO_x_ thin films are highly active and could accelerate the decomposition of the relevant PSC devices. So far, various strategies have been employed to address the issues; however, it is difficult to control whether the modifying molecules could be beneficial for achieving uniform coverage and stable adhesion [166, 167]. For large‐area fabrication of NiO_x_‐based PSC devices, CBD struggles to ensure uniform thickness of NiO_x_ thin films, often leading to the formation of the pinholes within the thin films or the areas with uneven thicknesses, which poses a significant obstacle to industrial applications of PSCs [53, 161].

#### 4.4.3 Relevant PSC Performance Optimization

It should be noted that, even though the CBD method has some issues that should be well addressed, this process, as a type of important wet chemical preparation method, also exhibits unique advantages in the precise structural regulation and low‐cost fabrication of NiO_x_ HTLs. Encouragingly, several strategies have so far been developed to address the existing challenges, mainly focusing on structural regulation, optimization of growth kinetics, and interface engineering. These strategies could be beneficial for achieving a leap in PSC device performance through precise regulation of the microstructure and electrical properties of NiO_x_ thin films. Compared with the precursor polycondensation mechanism of the sol–gel method and the self‐propagating reaction of the solution combustion method, the CBD method, through the slow release of ions in the liquid phase and controllable deposition, could enable easier realization of hierarchical structure design and uniformity regulation of thin films.

##### 4.4.3.1 Structural Regulation: Porous Morphology Optimizes Physical Contact

Sun et al. regulated the CBD process by introducing potassium persulfate, inducing the formation of a bilayer NiO_x_ structure with a dense bottom and ridge‐like mesoporous top [154]. Vertically continuous flakes of the obtained NiO_x_ bilayer, acting as anchoring points, significantly increased their interfacial contact area with the perovskite active layers (Figure 16a), leading to the reduced interfacial recombination kinetics (with extended *V*_OC_ decay lifetime), an increased *FF* value from 73% to 85%, and an improved PCE of 16.7%.

##### 4.4.3.2 Growth Kinetics Regulation: Obtaining Dense and Uniform Films

During the preparation of NiO_x_ HTLs via the CBD method, the anisotropic growth of nickel (Ni)‐based intermediate phases could tend to result in NiO_x_ thin films with a porous structure and poor uniformity. Furthermore, their electronic properties are difficult to effectively regulate; this issue directly restricts the improvement of the PCE values of the PSC devices and their long‐term stability. To address this bottleneck, Sheng et al. focused on the regulation of growth kinetics by introducing NH_4_Cl as an additive during the process [159]. By turning the concentration of [Ni(H_2_O)_x_(NH_3_)_6‐x_]^2+^ (Figure 16b), the authors inhibited the anisotropic growth of nickel (Ni)‐based intermediates, obtaining dense and uniform NiO_x_ thin films (with a roughness <8 nm). Combined with annealing treatment at 360°C to optimize the Ni^3+^/Ni^2+^ ratio (3.35), the offset of valence band energy level of the obtained NiO_x_ thin films was reduced from −0.91 eV to −0.21 eV, which effectively lowered the charge transport loss. Based on this optimization strategy, the performance of *p‐i‐n* PSC devices was significantly improved; that is, the champion PCE of MAPbI_3_‐based PSC could reach up to 19.75%, while that of (FA_0.98_MA_0.02_)_0.95_Cs_0.05_Pb(I_0.95_Br_0.05_)_3_‐based PSC was even as high as 23.30%. Moreover, the PCE of the 14‐cm^2^ mini‐module device also reached up to 19.36%, demonstrating excellent potential for large‐scale device application. Xu et al. employed triisopropanolamine (TPA) as a ligand; the strong coordination interaction of TPA with Ni^2+^ delays the ion release and promotes the in situ formation of dense Ni(OH)_2_ precursors [160]. After the annealing treatment, the films are rich in hydroxyl groups, providing sufficient anchoring sites for MeO‐2PACz. Due to the above, the interfacial non‐radiative recombination voltage loss could be reduced to 70 mV, and the relevant PSC device efficiency (Figure 16c) reaches up to 26.53% (certified 26.44%).

##### 4.4.3.3 Interface and Doping Engineering: Optimizing Energy Level Alignment, Crystallization, and Conductivity

**FIGURE 17:**
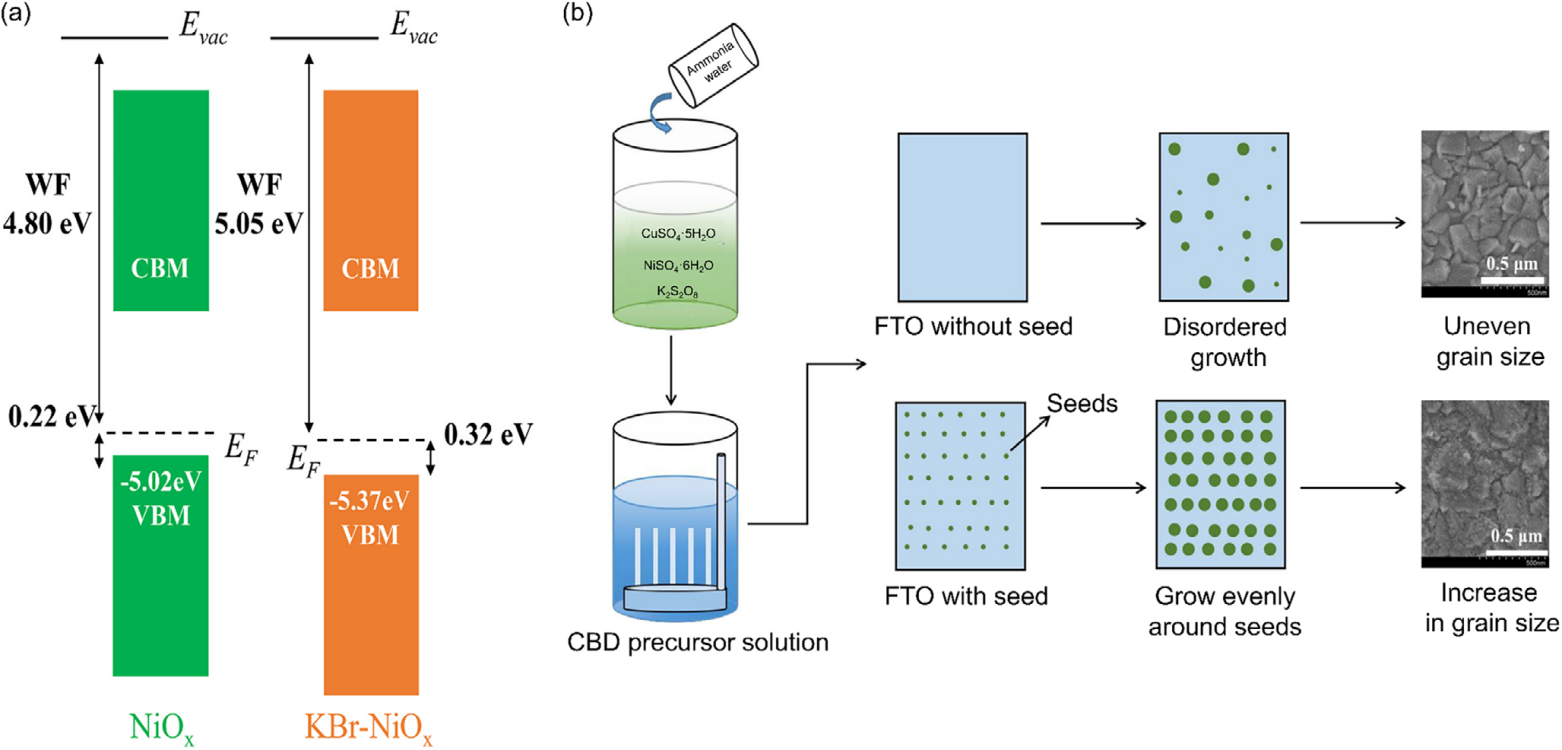
(a) Energy level scheme for the pristine and KBr deposited HTL, in which the parameters are derived from UPS spectra. Reproduced with permission [163]. Copyright 2023, Elsevier. (b) Schematic diagrams of the CBD process for the deposition of NiO_x_ films and the seeding‐induced crystallization by NiO_x_ seeds are presented. Reproduced with permission [18]. Copyright 2023, Wiley‐VCH.

Interface modification and doping strategies have also been employed to further enhance the carrier transport properties and stability of NiO_x_ thin films via using the CBD method. Dong et al. inserted a KBr buffer layer between NiO_x_ and perovskite layers, which shifted the VBM of NiO_x_ down from −5.02 eV to −5.37 eV (Figure 17a), optimizing energy level alignment within the devices [163]. Meanwhile, the diffusion of K^+^ ions reduced the activation energy for perovskite crystallization, promoting grain growth (average grain size increased from ∼150 nm to 172 nm) and enhancing film crystallinity, which effectively passivated intrinsic defects (e.g., undercoordinated Pb^2+^ sites and iodine vacancies) and reduced the defect density by 27%. These synergistic effects improved the PCE of the inverted PSC from 17.56% to 19.21%. Moreover, the unencapsulated devices retained 80% of their stability after 1100 h. Liao et al. further optimized the CBD method via combining seed‐assisted growth (SCBD) with Cu doping strategy (Figure 17b) [18]. Spin‐coated NiO_x_ nanocrystals were used as seeds to reduce the nucleation barrier of Ni(OH)_2_, promoting the growth of NiO_x_ grains, reducing grain boundary defects and pinholes, and achieving better substrate coverage; meanwhile, Cu ions (with ionic radius similar to Ni^2+^) were doped to form shallow acceptor levels in NiO_x_, which improved the film conductivity by ∼28% (surface current increased from 6.17 to 7.94 nA) and optimized the energy level alignment. The VBM of SCBD Cu:NiO_x_ reached 5.31 eV, closer to the VBM of perovskite (5.44 eV) to reduce energy loss during hole transport. Such synergistic effect could boost the PCE of the related PSCs from 18.13% to 22.51%, and the PCE of large‐area modules reached 19.36%, verifying the great potential of this method for large‐scale PSC device applications.

Even though the CBD method still has some aspects requiring optimization in the process of preparing NiO_x_ HTLs, as can be seen from the series of research work summarized above, the synergistic improvements of device efficiency and stability have been achieved through optimization strategies focusing on regulating interfacial contact, energy level alignment, and carrier transport kinetics. Through the strategies such as the construction of mesoporous structures, ligand‐regulated growth kinetics, ion doping, and interface buffer layer design, NiO_x_ HTLs have been further optimized from multiple dimensions, including microstructure (grain size, roughness), electronic properties (energy level alignment, conductivity), and interfacial properties (defect density, recombination rate). These strategies on NiO_x_ HTLs have enabled the synergistic improvement of the efficiency (from 16.7% to 26.53%) and stability of PSCs, with excellent potential for large‐scale PSC device applications. These efforts provide a systematic solution for the preparation of high‐quality NiO_x_ HTLs via the CBD method, beneficial for high‐performance, high‐stability, and large‐scale PSCs.

## 5 Future Strategies for Further Optimization

As detailed in the above sections, NiO_x_ thin films process unique characteristics, making them serve as promising HTLs for high‐performance and high‐stability PSC devices and holding great potential for large‐scale and/or flexible photovoltaic device applications. Beyond the specific technical challenges of each solution‐processed method, their compatibility with large‐area, high‐throughput deposition techniques (e.g., slot‐die coating, roll‐to‐roll) must be critically assessed against the industrial trade‐off between film uniformity and manufacturing throughput/cost. Among the four mainstream routes, pre‐synthesized NiO_x_ nanocrystal inks offer the most balanced solution: they enable room‐temperature deposition with excellent dispersion stability and batch‐to‐batch reproducibility, eliminating the need for in‐situ high‐temperature annealing [78, 92]. Solution combustion synthesis, despite its low thermal budget (∼150°C), suffers from precursor instability and gas evolution during film formation, which induces pinholes and narrows the processing window for continuous coating [133, 148]. Sol–gel processed NiO_x_, while mature in doping versatility, is fundamentally constrained by its high annealing temperature requirement (>300°C) [114], imposing high energy costs and prohibiting flexible substrate compatibility. Chemical bath deposition (CBD) excels in film conformality and buried interface control, particularly on textured FTO [154], yet its intrinsically slow deposition rate and wet chemical waste challenge high‐throughput manufacturing. Therefore, from an industrial scalability perspective, NiO_x_ nanocrystal inks represent the most pragmatic pathway. The core merit of solution‐based NiO_x_ synthesis lies in its low cost and facile equipment requirements. Future optimization should adhere closely to this core, overcoming bottlenecks within the framework of existing solution processes while avoiding reliance on high‐end equipment or additional costs to facilitate industrial implementation. As detailed above, we highlighted the experimental processes and working mechanisms for four types of main solution‐processed methods developed so far, including pre‐synthesized nanoparticle method, sol–gel method, solution combustion method, and chemical bath deposition, for synthesizing NiO_x_ thin films as HTLs in high‐performance PSC device applications. In addition, we have systematically discussed the existing intrinsic and/or technical challenges for each type of solution‐processed method for the syntheses of NiO_x_ thin films working as HTLs and summarized the main strategies that have been proposed to date to address the particular issues for each type of method.

**FIGURE 18:**
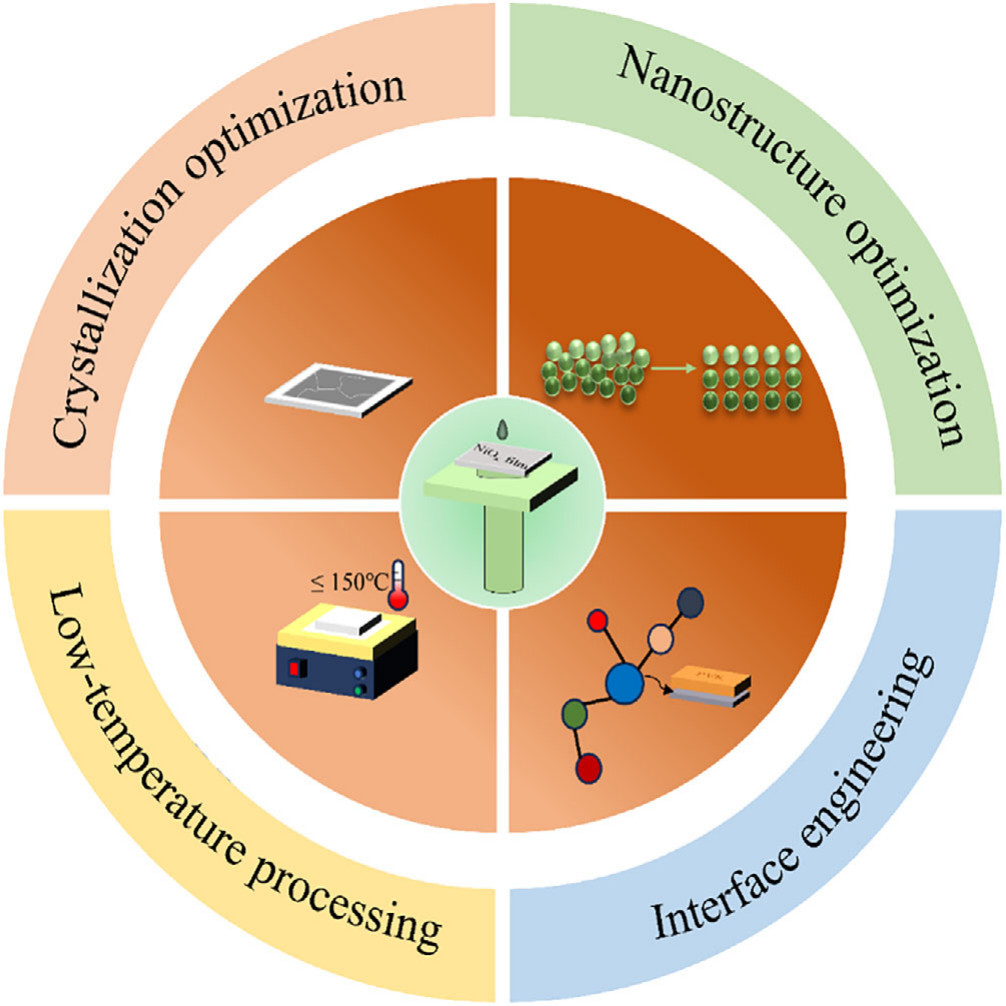
Schematic diagram of future research directions and advances concerning the solution‐prepared NiO_x_ thin films working as HTLs.

In addition to the particular issues of each type of solution‐processed method for synthesizing and optimizing NiO_x_ thin films, it should be noted that there is still room and some unsolved problems for further promoting the usage of solution‐processed NiO_x_ thin films as HTLs for high‐performance, high‐stability, and large‐sized PSC device applications, accelerating their commercialization. Future advances concerning the solution‐prepared NiO_x_ thin films working as HTLs are expected to be achieved in several exciting directions, such as crystallization optimization, novel low‐temperature processing techniques, nanostructure optimization, and interface engineering (Figure 18).

1. As for the above‐discussed four types of solution‐processed methods, some strategies have been developed to improve the quality of NiO_x_ thin films, including using metal cation doping or constructing the related bilayer. It should be noted that more novel strategies can be proposed to further reduce the defects and traps of the obtained NiO_x_ thin films prepared by these four types of solution‐processed approaches. Promisingly, defect regulation can focus on the integrated management within the solution system. By incorporating components with both doping and passivation functions into the precursor, carrier concentration and interfacial defects can be simultaneously optimized during film formation. Guided by conventional characterization techniques for formulation adjustment, this method eliminates the need for additional defect repair processes, reducing complexity.
2. The in‐depth and detailed understanding of the interfaces between perovskite active layers and NiO_x_ thin‐film HTLs will be of key importance for improving the performance and stability of PSCs. Strict synthesis protocols that specify details such as the control of ambient exposure must be developed and followed. As for optimizing interfacial compatibility, NiO_x_ energy levels can be tuned via solution‐coated buffer layers or low‐cost doping. Combined with theoretical calculations to screen suitable processes, this strategy would enable the flexible adaptation to systems such as tin (Sn)‐based and lead (Pb)‐free double perovskites, without relying on vacuum deposition equipment.
3. The new morphologies and forms of NiO_x_ thin films, such as nanostructures, should be thoroughly explored for the development of the next generation of PSCs. The nanostructured NiO_x_ thin films could offer tunability in multiple aspects, including quantum‐confined bandgaps, large surface areas, and doping densities. In addition, the mesoporous charge transport layers and nanorod/nanowire NiO_x_ thin films can enhance hole extraction and block electron transport, but the efficient infiltration of the active materials remains a major challenge. It would be necessary to further investigate these nanostructured NiO_x_ thin films and explore the charge carrier behaviors at their interfaces with perovskite active layers. Notably, the oriented nanorods/nanowires consisting of metal oxide materials can promote charge carrier transport and also suppress charge carrier recombination. It would be a promising pathway to explore the oriented NiO_x_ nanorods or nanowires through solution‐processed methods and to use them as efficient HTLs, thus beneficial for controlling and improving performance and stability of the PSC devices.
4. Furthermore, in addition to the above‐discussed four types of solution‐processed methods, new but reliable low‐cost solution processing methods will need to be developed for the growth of NiO_x_ thin‐film HTLs with high quality and promising physical features at low synthesis temperatures. In particular, it would be highly rewarding to conduct the related studies on the development of low‐temperature solution‐processed approaches for synthesizing high‐quality NiO_x_ thin films as efficient HTLs, which could enable the fabrication of high‐performance PSC applications entirely at room temperature. In terms of greenization and cost control, high priority should be given to low‐toxicity and easily accessible raw materials. Simple precursor recycling systems should be established to extend the storage life of precursors, thereby reducing waste and environmental risks.

In summary, future optimization would not only be based on the existing four types of solution processes but also encourage the development of new and novel solution‐based synthesis methods. Through low‐cost regulation of processes, precursors, defects, and interfaces, the advantages of using solution‐processed high‐quality NiO_x_ thin films as promising HTLs can be maximized to adapt to large‐scale industrial production of PSC devices.

## 6 Conclusion and Outlook

In this review, we systematically summarize the research progress, core advantages, and existing challenges of solution‐based methods for the synthesis of NiO_x_ thin films, focusing primarily on their application as the HTLs in the inverted PSC devices. We highlight and analyze the main challenges and issues for each type of solution‐processed synthesis method and systematically summarize and discuss the main strategies and approaches that have been developed so far to resolve these existing issues. Beyond, we propose further strategies for optimizing NiO_x_ thin films as the HTLs in the high‐performance, highly stable, and low‐cost PSC devices, aiming to further move this field forward.

As shown in Table 1, solution‐processed NiO_x_ HTLs, leveraging their significant advantages such as promising hole transport property, low cost, high stability, easy scalability, and simple equipment requirement, have seen continuously improved PSC device efficiency with impressive achievements, emerging as a key technical pathway driving the commercialization of PSCs. These techniques all rely on liquid‐phase reactions for the fabrication of NiO_x_ thin film, thus avoiding the high energy consumption and equipment complexity of traditional processes (e.g., vacuum sputtering, high‐temperature annealing). Through sustained innovation in materials and processes, the usage of solution‐processed NiO_x_ as HTLs has enabled PSCs to achieve the PCE from <20% [71, 126, 127, 133, 154] in the early stage to recently surpassing 27% in single‐junction devices and exceeding 30% in tandem cells [3, 4, 5, 102].

**TABLE 1:** Summary of the performance of single‐junction devices (PCE > 20%) and tandem devices mentioned in this review.

Year	Author	Structure	Method	PCE (Area)	Reference
2024	Song et al.	ITO/NiO_x_/perovskite/PCBM/BCP/Ag	Pre‐synthesized nanoparticle	23.27% (0.05 cm^2^)	[89]
2023	Liu et al.	FTO/NiO_x_/Me‐4PACz/perovskite/C_60_/BCP/Ag	Pre‐synthesized nanoparticle	25.1% (0.05 cm^2^)	[91]
2025	Zhang et al.	ITO/Mg:NiO_x_/Me‐4PACz/ PVK/PCBM/BCP/Ag	Pre‐synthesized nanoparticle	25.86% (0.06 cm^2^)	[88]
2025	Cheng et al.	FTO/NiO_x_/Me‐4PACz+AG/ PVK/C_60_/BCP/Ag	Pre‐synthesized nanoparticle	26.21% (0.09 cm^2^)	[81]
2025	Qu et al.	FTO/NiO_x_/Me‐4PACz/ PVK/C_60_/BCP/Ag	Pre‐synthesized nanoparticle	26.39% (0.08 cm^2^)	[98]
2024	Liu et al.	ITO/NiO/SAMs/FA_0.95_Cs_0.05_PbI_3_/PI/PC_61_BM/BCP/Ag	Pre‐synthesized nanoparticle	26.54% (0.06 cm^2^)	[97]
2025	Xiong et al.	FTO/NiO_x_/Me‐4PACz/FAPbI_3_/passivators/C_60_/SnO_2_/Ag	Pr e‐synthesized nanoparticle	27.2% (0.07 cm^2^)	[5]
2025	Liu et al.	ITO/NiO_x_/SAMs/perovskite/PCBM/BCP/Ag	Pre‐synthesized nanoparticle	27.27% (0.05 cm^2^)	[3]
2024	Kuo et al.	FTO/NiO_x_/MAPbI_3_/PC_61_BM/BCP/Ag	Sol‐gel	21.43% (0.04 cm^2^)	[131]
2018	Liu et al.	FTO/NiO_x_/MA_1−y_FA_y_PbI_3−x_Cl_x_/PCBM/BCP/Ag	Solution combustion	20.2% (0.08 cm^2^)	[148]
2024	Huang et al.	ITO/NiO_x_/perovskite/C_60_/BCP/Ag	Solution combustion	26.09% (0.06 cm^2^)	[153]
2023	Liao et al.	FTO/SCBD Cu:NiO_x_/perovskite/C_60_/BCP/Au	CBD	22.51% (0.06 cm^2^)	[18]
2024	Sheng et al.	FTO/ NiO_x_/perovskite/spiro‐OMeTAD/Ag	CBD	23.3% (14 cm^2^)	[159]
2025	Xu et al.	FTO/NiO_x_/MeO‐2PACz/perovskite/C_60_/BCP/Ag	CBD	26.44% (0.09 cm^2^)	[160]
2025	He et al.	ITO/NiO_x_/4PADCB+Ph‐4PACz/WBG perovskite (1.77 eV)/PiPBr/LiF/C_60_/BCP/Cu	Pre‐synthesized nanoparticle	20.35% (0.05 cm^2^)	[100]
2025	He et al.	ITO/NiO_x_/4PADCB+Ph‐4PACz/WBG perovskite/ALD SnO_2_/IZO/LBG perovskite/PEDOT:PSS/C_60_/BCP/Cu	Pre‐synthesized nanoparticle	27.03% (0.05 cm^2^)	[100]
2025	Luo et al.	ITO/NiO_x_/Me‐4PACz/ALB/WBG perovskite (1.67 eV)/C_60_/BCP/Ag	Pre‐synthesized nanoparticle	22.68% (/)	[104]
2025	Luo et al.	Top cell: MgF_2_/Glass/ITO/NiO_x_/Me‐4PACz/ALB/WBG perovskite (1.67 eV)/C_60_/SnO_2_/ITO Bottom cell: Glass/Mo/CIGS/CdS/i‐ZnO/AZO	Pre‐synthesized nanoparticle	29.06% (/)	[104]
2025	Yang et al.	Top cell: ITO/NiO_x_/Me‐4PACz/WBG perovskite (1.78 eV)/PDAI_2_/PCBM/SnO_2_/ITO Bottom cell: ITO/PEDOT: PSS/2D Seeds/Pb‐Sn perovskite (1.23 eV)/PCBM/BCP/Ag	Pre‐synthesized nanoparticle	27.68% (0.05 cm^2^)	[105]
2025	Luo et al.	Top cell: ITO/NiO_x_/PyAA‐MeO SAM/WBG perovskite/C_60_/SnO_2_/IZO Bottom cell: Si with planar front and textured back	Pre‐synthesized nanoparticle	30.9% (1 cm^2^)	[102]
2025	Shi et al.	ITO/NiO_x_/SAM/WBG perovskite (1.77 eV)/PDAI_2_/C_60_/BCP/Ag	Pre‐synthesized nanoparticle	20.21% (0.09 cm^2^)	[101]
2025	Shi et al.	ITO/NiO_x_/SAM/WBG perovskite (1.77 eV)/PDAI_2_/C_60_/SnO_2_/Au/PEDOT: PSS/NBG perovskite (1.26 eV)/EDAI_2_/C_60_/BCP/Ag	Pre‐synthesized nanoparticle	28.78% (0.09 cm^2^)	[101]
2025	Fu et al.	ITO/NiO_x_/Me‐4PACz/WBG perovskite (1.77 eV)/PDAI_2_/C_60_/BCP/Cu	Pre‐synthesized nanoparticle	20.35% (0.07 cm^2^)	[106]
2025	Fu et al.	ITO/NiO_x_/Me‐4PACz/perovskite (1.56 eV)/PDAI_2_/C_60_/BCP/Cu	Pre‐synthesized nanoparticle	25.71% (0.07 cm^2^)	[106]
2025	Fu et al.	ITO/NiO_x_/Me‐4PACz/WBG perovskite (1.77 eV)/ C_60_/SnO_2_/Au/PEDOT: PSS/NBG perovskite/C_60_/BCP/Cu	Pre‐synthesized nanoparticle	28.13% (0.07 cm^2^)	[106]
2024	Duan et al.	ITO/NiO_x_/SAMs/WBG perovskite (1.68 eV)/C_60_/BCP/Cu	Pre‐synthesized nanoparticle	21.5% (1.05 cm^2^)	[110]
2024	Duan et al.	ITO/NiO_x_/SAMs/WBG perovskite (1.68 eV)/C_60_/ SnO_2_/Au/PEDOT: PSS/NBG perovskite (1.23 eV)/C_60_/SnO_2_/Ag	Pre‐synthesized nanoparticle	26.3% (1 cm^2^)	[110]
2024	Duan et al.	Top cell: ITO/NiO_x_/SAMs/WBG perovskite (1.68 eV)/C_60_/SnO_2_/IZO/Ag Bottom cell: Si	Pre‐synthesized nanoparticle	27.8% (1 cm^2^)	[110]
2024	Yang et al.	ITO/NiO_x_/SAMs/WBG perovskite/C_60_/ SnO_2_/Au/PEDOT: PSS/NBG perovskite (1.22 eV)/C_60_/SnO_2_/Ag	Pre‐synthesized nanoparticle	28.87% (0.04 cm^2^)	[111]
2025	Dong et al.	ITO/NiO_x_/2PACz/WBG perovskite (1.83 eV)/C_60_/BCP/Ag/MoO_3_/2PACz/PM6:D18:BTPeC9/C_60_/BCP/Ag	Pre‐synthesized nanoparticle	25.34% (0.06 cm^2^)	[113]
2025	Wang et al.	FTO/NiO_x_/MPA‐CPA/WBG perovskite (1.77 eV)/PCBM/PEI/SnO_x_/Ag	Pre‐synthesized nanoparticle	20.5% (0.25 cm^2^)	[107]
2025	Wang et al.	FTO/NiO_x_/MPA‐CPA/WBG perovskite (1.77 eV)/PCBM/PEI/SnO_x_/IZO/PEDOT:PSS/NBG perovskite (1.26 eV)/C_60_/SnO_x_/Ag	Pre‐synthesized nanoparticle	28.6% (0.25 cm^2^)	[107]
2024	Jia et al.	ITO/NiO_x_/Me‐4PACz/WBG perovskite (1.67 eV)/LiF/C_60_/BCP/Ag	Pre‐synthesized nanoparticle	22.78% (0.09 cm^2^)	[108]
2024	Jia et al.	ITO/NiO_x_/Me‐4PACz/WBG perovskite (1.79 eV)/LiF/C_60_/BCP/Ag	Pre‐synthesized nanoparticle	20.21% (0.09 cm^2^)	[108]
2024	Jia et al.	ITO/NiO_x_/Me‐4PACz/WBG perovskite (1.79 eV)/LiF/C_60_/SnO_2_/Au/PEDOT:PSS/NBG perovskite (1.25 eV)/C_60_/BCP/Ag	Pre‐synthesized nanoparticle	28.52% (0.09 cm^2^)	[108]
2025	Zheng et al.	ITO/SnO_2_/MgF_2_/C_60_/WBG perovskite (1.91 eV)/MeO‐2PACz/NiO_x_/SnO_2_/C_60_/perovskite (1.55 eV)/MeO‐2PACz/ITO/Si	Pre‐synthesized nanoparticle	27.06% (1 cm^2^)	[103]
2025	Pei et al.	ITO/NiO_x_/SAM/WBG perovskite (1.68 eV) +TAR 3/C_60_/BCP/Ag	Pre‐synthesized nanoparticle	23.5% (0.1 cm^2^)	[109]
2025	Pei et al.	ITO/NiO_x_/SAM/perovskite (1.53 eV) +TAR 3/C_60_/BCP/Ag	Pre‐synthesized nanoparticle	25.8% (0.1 cm^2^)	[109]
2025	Pei et al.	Mo/CIGS/CdS/ZnO/ITO/NiO_x_/SAM/WBG perovskite (1.68 eV)/C_60_/SnO_x_/ITO/MgF_x_/Ag.	Pre‐synthesized nanoparticle	28.05% (0.21 cm^2^)	[109]
2025	Lin et al.	ITO/NiO/SAM/WBG perovskite (1.78 eV)/C_60_/ALD‐SnO_2_/Au/PEDOT:PSS/NBG perovskite (1.25 eV)/C_60_/BCP/Cu	Pre‐synthesized nanoparticle	30.1% (0.05 cm^2^)	[4]
2023	Tyagi et al.	Top cell: ITO/cp‐NiO_x_/perovskite (1.68 eV)/PC_60_BM/ZnO/Ag Bottom cell: Si	Sol‐gel	26.01% (/)	[130]

It is worth noting that the current inverted structure PSCs that achieve high efficiency (as listed in Table 1) generally adopt the NiO_x_/SAMs composite hole transport structure. The high‐quality NiO_x_ layer first acts as an ideal substrate, which, through optimized synthesis and doping (such as increasing the proportion of Ni^3+^ and regulating conductivity), provides the device with efficient intrinsic hole transport capability, excellent film morphology, and chemical stability [18, 56, 57]. More importantly, the abundant metal sites on the NiO_x_ surface and hydroxyl (─OH) groups significantly improve the assembly quality of subsequent SAMs, enabling them to form denser, more uniform, and more firmly bonded molecular layers, solving the problems of uneven coverage, poor thermal stability, etc. when SAMs are directly assembled on TCO [80, 168]. On this basis, the carefully designed SAMs (such as Me‐4PACz) play a key role at the interface, including precisely regulating the interface energy level alignment, passivating the interface defects between NiO_x_ and the perovskite (such as uncoordinated Ni ions, Pb^2+^ vacancies), and optimizing the bottom crystallization environment of the perovskite [78, 131, 132]. Therefore, NiO_x_ provides a stable hole transport and stable substrate foundation, while SAMs are the key to achieving extreme interface passivation and energy level optimization, and the two complement each other, jointly forming the cornerstone for achieving a >26% high efficiency.

Despite the notable progress in solution‐processed NiO_x_ HTLs for PSCs, to fully unlock their industrialization potential, future efforts need to focus on in‐depth research and understanding into the solution rheology, composite doping, development of more advanced solution‐processed coating technologies (e.g., slot‐die coating, inkjet printing, flexographic printing), and optimization of drying/crystallization kinetics to achieve large‐area, high‐speed, high‐uniformity, and high‐yield fabrication of high‐quality NiO_x_ thin films. While progress has been made in low‐temperature processing, some high‐performance strategies (such as certain doping approaches and high crystallinity requirements) still rely on specific temperatures (>150°C) or post‐treatments (e.g., UV treatment). Investigating solution processes that can achieve high conductivity, excellent energy level alignment, low defect state density, and good interfacial contact under near room temperature (≤100°C) or even annealing‐free conditions is crucial for meeting the demands of ultra‐flexible and ultra‐low‐cost manufacturing of PSC devices.

All in all, leveraging the advantages in facile processing, low cost, and high hole transport features, solution‐processed NiO_x_ HTLs are expected to take the lead in commercialization in scenarios such as flexible photovoltaics and building‐integrated energy applications. Through deepened mechanistic understanding and technological innovation in the future, NiO_x_‐HTL‐based PSC devices will undoubtedly become a vital force in the photovoltaic industry.

## Author Contributions

Z. Qiu and F. Li conceptualized this work, proposed the overall logical framework of the review and critically revised the manuscript. Z. Wu collected and analyzed the relevant literature and drafted the manuscript. Y. Tao and Y. Qiu assisted in collecting and organizing the relevant literature. Y. Duan provided writing advice and assisted with manuscript revisions. Q. Peng revised the manuscript.

## Conflicts of Interest

The authors declare no conflicts of interest.

## Data Availability

Data sharing is not applicable to this article as no datasets were generated or analyzed during the current study.
